# Identification of the cross-strand chimeric RNAs generated by fusions of bi-directional transcripts

**DOI:** 10.1038/s41467-021-24910-2

**Published:** 2021-07-30

**Authors:** Yuting Wang, Qin Zou, Fajin Li, Wenwei Zhao, Hui Xu, Wenhao Zhang, Haiteng Deng, Xuerui Yang

**Affiliations:** 1 MOE Key Laboratory of Bioinformatics, Center for Synthetic & Systems Biology, School of Life Sciences, Tsinghua University, Beijing, China; 2 Joint Graduate Program of Peking-Tsinghua-National Institute of Biological Science, Beijing, China

**Keywords:** Bioinformatics, Data mining, Transcriptomics

## Abstract

A major part of the transcriptome complexity is attributed to multiple types of DNA or RNA fusion events, which take place within a gene such as alternative splicing or between different genes such as DNA rearrangement and trans-splicing. In the present study, using the RNA deep sequencing data, we systematically survey a type of non-canonical fusions between the RNA transcripts from the two opposite DNA strands. We name the products of such fusion events cross-strand chimeric RNA (cscRNA). Hundreds to thousands of cscRNAs can be found in human normal tissues, primary cells, and cancerous cells, and in other species as well. Although cscRNAs exhibit strong tissue-specificity, our analysis identifies thousands of recurrent cscRNAs found in multiple different samples. cscRNAs are mostly originated from convergent transcriptions of the annotated genes and their anti-sense DNA. The machinery of cscRNA biogenesis is unclear, but the cross-strand junction events show some features related to RNA splicing. The present study is a comprehensive survey of the non-canonical cross-strand RNA junction events, a resource for further characterization of the originations and functions of the cscRNAs.

## Abstract (web summary)

Gene fusion, trans-splicing or transcription read-through contributes to generation of chimeric RNA. Here the authors develop a pipeline to identify non-canonical type of chimeric RNAs called cross-strand chimeric RNA (cscRNA), which are fused between two precursor RNAs transcribed from the opposite DNA strands.

## Introduction

It has been increasingly recognized that the genetic and epigenetic information is subjected to complicated processing during and after transcription, which gives rise to the extensive transcriptome complexity^1,2^. Variations in RNA splicing, which result in different isoforms of the mature RNA products from the same gene, is a major machinery contributing to the transcriptome complexity in the species of metazoans^3,4^. In addition, gene fusion generates hybrid RNA products, i.e., chimeric RNAs, and brings the transcriptome complexity to a higher level^5–7^.

Chimeric RNAs could be results of chromosome rearrangement at the DNA level or non-canonical RNA splicing at the RNA level^8^. Gene fusion due to chromosome rearrangement is a well-characterized hallmark of cancer^9^, which is infrequent in the non-cancerous contexts^10^. Many of such gene fusion events give rise to fusion proteins that often play critical roles in cancer development, maintenance, and progression^11^. Therefore, several fusion proteins, for example, BCR-ABL1 in chronic myelogenous leukemia^12^ and EML4-ALK in lung cancer^13^, serve as effective therapeutic targets^6,14,15^. Another type of gene fusion takes place between RNA molecules in the absence of DNA rearrangement, for example, trans-splicing or transcription read-through between two precursor RNAs^16^. Such non-canonical RNA splicing between different genes is another source of transcriptome complexity^17^, which has been frequently reported in both cancerous and normal cell contexts^10,18–23^. Examples of such RNA chimeras include JAZF1-SUZ12 (JAZF1-JJAZ1)^10^, PAX3-FOXO1^21^, and SLC45A3-ELK4^17,24^, which were shown to be physiologically relevant and subjected to sophisticated regulations on their prevalence.

In general, the trans-splicing that generates chimeric RNA and the back-splicing that produces circular RNA depend on spatial proximity between the RNA fragments to be fused together^25–27^. In many cases, such geometric closeness was mediated by base-pairing between the complementary sequences of the precursor RNAs^28,29^. Here, we reason that by definition, the overlapping bi-directional transcripts from the two opposite strands of the genome DNA within a limited region should share complementary sequences and should be geometrically close before leaving their transcription sites. Therefore, we ask whether these transcripts could form a different type of chimeric RNAs, which are cross-strand RNA fusion products, i.e., cross-strand chimeric RNAs (cscRNAs).

Several bioinformatics pipelines have been developed to search for the chimeric RNA transcripts with RNA sequencing data^30–34^. Their main aims are mostly identifications of the gene fusion events between the annotated transcripts. Therefore, although thousands of chimeric RNA species have been identified^8,35–37^, including small numbers of cscRNAs formed between previously known transcripts, none of the current methods can perform de novo identification of the cscRNAs in an unbiased and comprehensive manner. In the published databases for the gene fusion events, the category of cscRNA has been largely overlooked, except for ChiTaRS, which, to our best knowledge, is the only database hosting an archive of the cross-strand fusion transcripts^36^. In fact, the chimeric RNA species in ChiTaRS were identified from the ESTs and mRNAs from GenBank. These sequences from different studies were all pooled together, and the context information is lost. Here we designed a specialized bioinformatics pipeline cscMap for de novo identification of the cross-strand chimeric RNAs directly from the RNA-seq reads, in a context-specific manner. A series of measurements were carefully implemented by cscMap to obtain high accuracy for capturing the cross-strand junction events. We used the RNA deep sequencing data from a large variety of biological samples in the ENCODE consortium and the GEO database. Interestingly, large numbers of cscRNAs were identified in both the normal and cancerous contexts, and they were found in multiple species as well. Experimental validations and other features of these chimeric RNAs suggested that most of them are not products of genome rearrangement and that they are unlikely to be just technical artifacts or biological noise. These cross-strand RNA fusion events predominantly take places between products of the bi-directional convergent transcriptions. Further analyses then suggested that these cross-strand RNA junctions are potentially related to RNA splicing and possibly other transcriptional and post-transcriptional processes. Although the detailed machinery of cscRNA biogenesis and their potential functions are still unclear, we propose that they represent an additional level of the transcriptome complexity, which is worth further investigation. The present study hereby provides a comprehensive survey of the cscRNAs in a large variety of samples, which should serve as an insightful resource to inspire and support further elucidation of the biological relevance of the cscRNAs.

## Results

### Identification of the cscRNA species with paired-end RNA-seq data

**Fig. 1:**
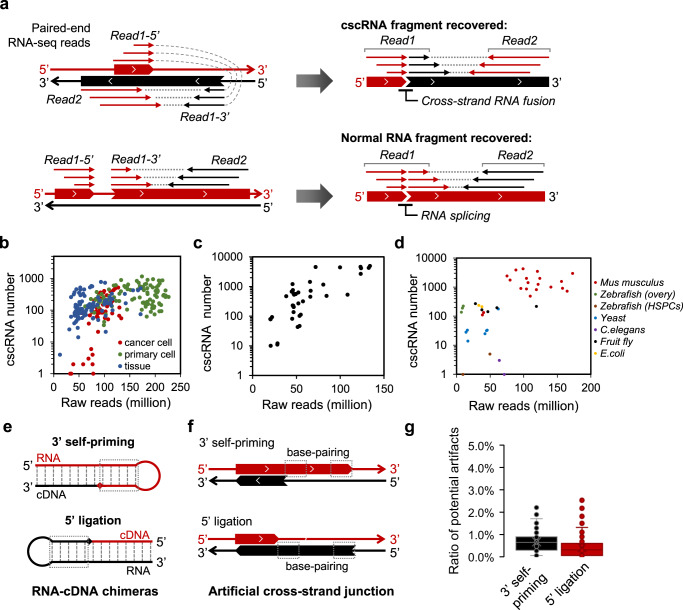
Identification of the cscRNAs with total RNA PE sequencing datasets. **a** Alignment scenarios of the PE RNA-seq reads for identification of the cross-strand RNA fusion events (top) and the regular exon–exon junctions upon RNA splicing (bottom). Top: one end (e.g., read1) of the PE reads were truncated into two fragments, which were mapped to the two opposite reference strands, respectively. The other end (read2) was then mapped to the shifted strand accordingly. Bottom: one end (e.g., read1) of the PE reads were truncated into two fragments, which were mapped to two positions of the same strand, indicating an exon–exon junction upon RNA splicing. The other end (read2) was mapped to the same strand. **b** The numbers of cscRNAs identified in each of the human RNA-seq datasets from ENCODE, including 54 samples of cancer cell lines, 109 samples of primary cells, and 108 samples of normal tissues. The raw read number of each dataset is shown on the X axis. **c** The numbers of cscRNAs identified in each of the human RNA-seq datasets from the GEO database. The raw read number of each dataset is shown on the X axis. **d** The numbers of cscRNAs identified in the datasets of multiple other species, including *mouse* (20 samples), *zebrafish* (5), *C. elegans* (6), *fruit fly* (5), *yeast* (7), and *E. coli* (2). The datasets of mouse are from ENCODE, and all the others are from the GEO database. The raw read number of each dataset is shown on the X axis. **e** Potential artifacts during reverse transcription due to 3′ self-priming (top) of the template RNA or 5′ ligation (bottom) between the template RNA and the cDNA, both of which generate RNA–cDNA chimeras. **f** Artificial cross-strand junction events recovered by the RNA-seq reads of the RNA–cDNA chimeras shown in (**a**). The dashed boxes illustrate the reverse-complementary regions on the same strand, which strongly indicate the potential artifacts during reverse transcription as shown in (**a**). **g** Box plots illustrating the percentages of the potential artificial cscRNAs that show signatures of 3′ self-priming or 5′ ligation, in the 271 human samples from ENCODE. As shown in (**b**), base-pairing for at least 4 bp in the upstream fragment is deemed as a sign of 3′ self-priming, whereas base-pairing in the downstream fragment is a sign of 5′ ligation. The distance between the base-pairing regions was set to be within 100 nt. The median value is shown as the line and the average as the cross. Source data are provided as a Source data file.

We developed a bioinformatics pipeline cscMap to search for the cscRNAs, i.e., RNA chimeras resulted from fusions of the transcripts encoded by the two opposite DNA strands (Fig. 1a). While most of the current methods for identification of the chimeric RNA transcripts capture the gene fusion events between the annotated transcripts, cscMap was specifically designed for de novo identification of the non-canonical cross-strand junction events between the annotated or unannotated RNA transcripts. Briefly, paired-end (PE) RNA-seq reads were aligned to the reference genome and transcriptome by following the common practice. The un-mapped reads were then used for a second-round mapping, which breaks down one end of a PE RNA-seq read (i.e., read1 or read2) into two fragments and seeks alignments of these fragments to the two opposite DNA strands, respectively (Figs. 1a, S1). In addition, as a critical support to the fragmented and strand-shifted mapping of this one end, the other end of the PE read was used for mapping to the shifted strand (Figs. 1a, S1). The PE RNA-seq reads extracted by the process above were then used to recover the cscRNA sequences around the cross-strand RNA fusion sites. Finally, to eliminate the potential false discoveries, cscMap performs BLAST against the genome and transcriptome to remove the cscRNAs with sequence similarity to the normal genome or transcriptome (Fig. S1). In addition, the cscRNAs showing signs of potential artifacts during reverse transcription, such as RNA 3′ self-priming, 5′ ligation, or template switching, were also filtered out, which will be discussed in the following section. Technical details of the pipeline are provided in the “Methods”.

The pipeline was first applied on 271 datasets of total RNA PE sequencing generated by ENCODE, which offers advantages of high sequencing depths, large sample size, tissue type variety, and strand-specific sequencing with long paired-end reads. These are all human samples including 35 normal tissues, 52 primary cells, and 27 cancer cell lines, and each type of the samples has at least two biological replicates (Supplementary Data 1). For each sample, the cscRNAs supported by at least two PE reads were reported by cscMap. Detailed information of the cscRNAs identified by cscMap from these datasets is supplied in Supplementary Data 2. The numbers of cscRNA species in each sample and the total sequencing reads are summarized in Fig. 1b, showing a weak association in general. However, at similar sequencing depths, the numbers of cscRNAs could vary significantly across different samples, ranging from 10 s to about 1000 (Fig. 1b). Nevertheless, cscRNAs seem to be prevalence in most of the normal tissues, primary cells, and cancer cells. In total, 53,688 cscRNAs have been identified from these 271 samples combined. Among them, 3013 cscRNAs are recurrent in at least two samples. For each of these recurrent cscRNAs, the same cross-strand junction event takes place in multiple samples, all between exactly the same pairs of the nucleotide sites. Taking cscR-2-1 as an example, in total, 101 RNA-seq PE reads in 21 samples all support the same event of cross-strand junction. All the RNA-seq reads aligned to the two strands around the cross-strand junction sites of cscR-2-1are shown in Fig. S2.

Furthermore, cscMap has also identified large numbers of cscRNAs using many other RNA-seq datasets from the GEO database (Fig. 1c, information of the datasets is provided in Supplementary Data 1), which were generated by different labs with various types of human samples, suggesting that cscRNAs are unlikely to be technical artifacts just in the ENCODE data. 1815 of the total 53,688 cscRNAs or 724 of the 3013 recurrent ones identified with the ENCODE data were also found in these independent datasets. In addition, although the RNA-seq data of TCGA is not ideal for de novo identification of the cscRNAs due to the relatively short length and non-strand-specific reads, they provide direct supports to the cscRNAs that we identified with the ENCODE data. For example, for 80% (2420/3013) of the recurrent cscRNAs, their cross-strand junction sites were directly supported by the unmapped RNA-seq reads obtained from 100 randomly picked samples from the LUAD dataset in TCGA. Interestingly, various numbers of cscRNAs were identified in other species as well, such as *mouse*, *zebrafish*, *C. elegans*, *Fruit fly*, *yeast*, and *E. coli* (Fig. 1d, the cscRNAs identified in mouse samples are provided in Supplementary Data 3), indicating that the cross-strand junction is not limited to human. However, very few of the human cscRNAs (30 of the total 53,688 cscRNAs, Supplementary Data 2) were also found in mouse, which is not surprising. As a reference, the non-coding genome is known for its poor cross-species conservation^38–40^. Nevertheless, if we only look at the 5’ or 3’ junction sites of the cscRNAs, 378 (for the 5′ junction sites) and 735 (for the 3′) of them were also found in the mouse cscRNA library.

cscRNAs are frequently found in the normal tissues and primary cells, suggesting that the observed cross-strand fusion events, in general, are unlikely due to DNA recombination, which should be rare in non-cancerous samples. Nevertheless, we compared the cscRNAs to the known DNA rearrangement events in the gene fusion databases, such as FusionCancer and TCGA Fusion genes^41,42^. A very small proportion (0.3%, 391/120038) of the 5′ and 3′ junction sites were indeed consistent with the DNA recombination breakpoints. In addition, we used the DNA-seq data of 9 breast cancer cell lines (GSE48216)^43,44^, and only 0.6% (320/53688) of the cscRNAs or 0.9% of the recurrent ones were supported by the DNA-seq data (Supplementary Data 2).

### Technical artifacts for identification of the cscRNAs

It has been reported that when the 3′ end of an RNA molecule folds back and forms a terminal stem-loop structure, such self-priming of the RNA template could potentially result in elongation of the RNA at its 3′ end during reverse transcription (Fig. 1e, top)^45^. In another scenario, if the 5′ end of an RNA folds back and forms a stem-loop structure, it is possible that the cDNA sequence generated by reverse transcription would be ligated to the 5′ end of the RNA template (Fig. 1e, bottom)^45^. These two types of RNA–cDNA chimeras would then serve as templates for synthesis of the second strand cDNA, followed by PCR amplification and high-throughput sequencing. In both scenarios, the cDNA fragment of the RNA–cDNA chimera by definition should be reverse-complementary to a part of the original RNA molecule (Fig. 1e). These RNA–cDNA chimeras would appear to be synthesized from the two strands of the genome DNA as templates, therefore, serving as sources of artificial cscRNAs (Fig. 1f). However, these cscRNAs by definition should exhibit special sequence patterns as described above and shown in Fig. 1e, f, which allowed us to precisely identity these potentially false discoveries from the large pool of cscRNAs identified by cscMap. For each of the 271 samples from ENCODE, only a minimal proportion of the cscRNAs (usually <1%) showed features of possible 3′ self-priming or 5′ ligation (Fig. 1g), both of which were very loosely defined as terminal stem structures with just 4 or more base pairs. Therefore, the potentially false cscRNA species due to 3′ self-priming or 5′ ligation, if any, are trivial, and they have been removed during the filtering step of cscMap (Fig. S1).

Another potential source of the artificial cscRNAs is the rare events of template switching during reverse transcription, which have been strongly associated to short homologous sequences (SHSs) at the junction sites^28,46^. cscMap implemented a stringent filter to remove the cscRNAs with SHSs (>= 4 nt) around the junction sites. In addition, the sequencing read-mapping errors could also give rise to false discoveries of cscRNAs, even though cscMap only uses the regularly un-mappable RNA-seq reads to recover the cscRNA species. We tested whether the cscRNA reads can be mapped back to just one template strand of the genome with relatively low mapping scores. Most of these efforts failed, i.e., generally negligible proportions of the cscRNA reads could be re-aligned conventionally to just one strand (Fig. S3), which have been filtered out by cscMap (Fig. S1).

Furthermore, we generated testing RNA-seq data to benchmark the accuracy of cscMap in mapping the cross-strand junction reads and identifying the cscRNAs species. First, for the sensitivity of cscMap to the cross-strand junction reads, we used the real RNA-seq data in ENCODE supplied with simulated cross-strand junction reads from randomly designed cscRNAs. Similar tests were performed for 10 times, and cscMap exhibited very high sensitivity to these simulated cscRNA reads (Fig. S4a). Second, for the specificity of cscMap, we used Polyester to generate junction reads from the same DNA strand but unmappable to the reference transcriptome or fusion reads between sequences from different chromosomes^47^. These reads were supplemented into the real RNA-seq datasets as inputs of cscMap. As shown in Fig. S4b, very small and negligible proportions of these simulated junction or fusion reads were allocated by cscMap to support cross-strand junction events. Together, these results indicate high specificity and sensitivity of cscMap for allocating the cross-strand junction reads and identifying the cscRNAs.

### Abundance and tissue context-dependency of the cscRNAs

**Fig. 2:**
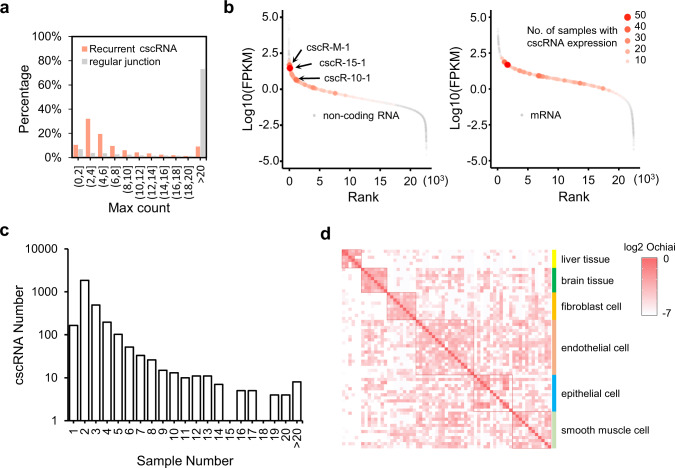
Expressions of the recurrent cscRNAs. **a** Distributions of the maximum RNA-seq read counts, among the 271 human samples from ENCODE, on the cross-strand junction sites of the recurrent cscRNAs or 10,000 randomly selected regular exon–exon junction sites. **b** The maximum of the FPKMs among the 271 human samples of the recurrent cscRNAs on the background of all the annotated long non-coding RNA (left) or mRNA (right) species. The size of each dot indicates the number of samples in which the cscRNA is expressed. The 3 most frequently found cscRNAs among all the samples are marked on the plot. **c** Distributions of the numbers of different biological samples in which the recurrent cscRNAs are expressed. **d** Similarity matrix showing the log2 Ochiai values based on the recurrent cscRNAs shared by some of the 271 human samples from ENCODE. Refer to Fig. S7 for the similarity matrix for all of the 271 samples.

With the technical artifacts ruled out, we then tested whether some of the cscRNA species are abundant in the cells and therefore unlikely to be just biological noise. Here we only looked at the 3013 recurrent cscRNAs from the 271 ENCODE samples. First, by taking the maximum among all the samples, the numbers of the RNA-seq reads covering the junction site of each cscRNA are distributed in a range slightly lower than that of 10,000 randomly selected regular exon–exon junctions (Fig. 2a). Such observation was confirmed by the read counts in each of the individual samples (three examples shown in Fig. S5a–c, including osteoblast, hair follicle dermal papilla cell, and smooth muscle cell of trachea, the top three samples with the largest numbers of cscRNAs). Next, we estimated the FPKMs of the cscRNAs based on the RNA-seq data. Note that due to the fragmentation process during library preparation of the RNA-seq assays, it is difficult to precisely recover the full length of the cscRNA species. Therefore, we just used the RNA-seq reads covering the junction sites as proxies to estimate the FPKMs of the cscRNAs. The cscRNA fragment lengths recovered by these PE reads were used to normalize the transcript lengths. Figure 2b shows the highest FPKMs of the cscRNAs and other coding and non-coding transcripts among all the samples. In general, the cscRNA are expressed in a broad range, just like the regular coding and non-coding RNA transcripts are. A small group of cscRNAs are relatively abundant, which are comparable to some annotated mRNAs and highly expressed long non-coding RNAs (Fig. 2b). This again was consistent with the analysis in each individual sample (three examples shown in Fig. S5d–f).

Next, we assessed the context-dependency of the cscRNAs by counting the overlapping cscRNAs across different samples. 94% of the recurrent cscRNAs were detected in at least two different types of the biological samples (Fig. 2c). Several hundreds of the cscRNAs were found in 5 or more types of different biological samples (Fig. 2c). The primary tissues and cells, which were sequenced to the highest depths, tend to have more of the recurrent cscRNAs (Fig. S6). Therefore, we believe that the tissue type-specificity of the cscRNAs is at least partly due to the low abundance of the cscRNAs and the limited sequencing depths. As mentioned above already, the stringent thresholds implemented by cscMap could have resulted in many false negatives, especially with relatively fewer sequencing reads. Nevertheless, as shown by the similarity matrix based on numbers of the common cscRNAs shared by different samples (Fig. 2d), the samples from the same organs or the biologically similar primary cells were clustered together, as they do share more common cscRNAs. Such examples include the liver tissues (such as liver, right lobe of liver), brain tissues (such as diencephalon, frontal cortex, occipital lobe, temporal lobe), and multiple types of primary cells such as fibroblasts, endothelial cells, etc. (Fig. 2d, Fig. S7). In summary, like what has been observed with the lncRNAs, the cscRNAs have broad-range and tissue-dependent expression profiles among the biological samples.

### Experimental validations and potential biological relevance of the cscRNAs

**Fig. 3:**
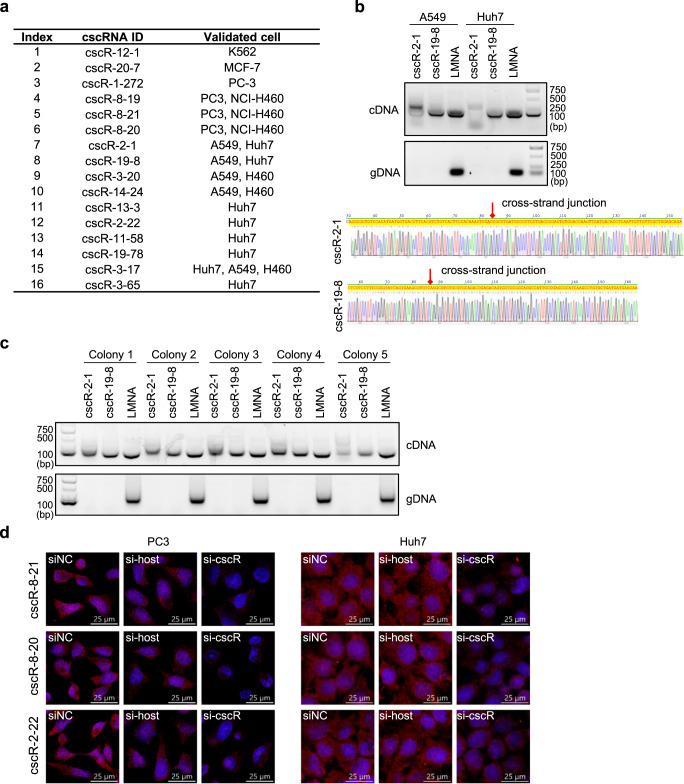
Experimental validations of the cscRNAs. **a** 16 cscRNAs were experimentally validated in different cell lines, by RT-PCR followed by Sanger sequencing. **b** As two examples, the sequences covering the cross-strand junction sites of cscR-2-1 and cscR-19-8 were amplified by PCR with the cDNA of A549 and Huh7 cells (top). The amplified sequences were subjected to Sanger sequencing (bottom). The Sander sequencing results of the other 14 cscRNAs are shown in Fig. S8. Source data are provided as a Source data file. **c** The sequences covering the cross-strand junction sites of cscR-2-1 and cscR-19-8 were amplified by PCR with the cDNA (top) or genomic DNA (bottom) from 5 cell colonies derived from A549 single cells. Representative images from three repeated experiments are shown. Source data are provided as a Source data file. **d** Representative images of RNA FISH, from three biological replicates, with the probes targeting cscR-8-21, cscR-8-20, or cscR-2-22 (red) in PC3 and Huh7 cells. The cell nucleus was stained with DAPI (blue). RNA knockdown with the siRNAs targeting the cscRNAs or the potential host gene transcripts were performed to show the specificity of the FISH probes.

A set of cscRNAs has been validated with PCR in multiple cancer cell lines (A549, Huh7, MCF7, PC3, K562, and NCI-H460) (Fig. 3a), even though many of these cscRNAs were initially not found in the particular cell lines used for the validations. In fact, given the low abundance of the cscRNAs (Supplementary Data 2) and the strict identification and filtering schemes that we have applied, it is very likely that cscMap has missed many cscRNAs with a given dataset, i.e., false negatives. Nevertheless, all of the PCR amplified products have been validated by Sanger sequencing, which directly confirmed the cscRNA sequences as identified by cscMap (examples given in Figs. 3b, S8).

Detections of the cscRNA sequences by PCR with the genome DNA from bulk cells were negative. However, it is still possible that the cscRNAs were originated from DNA recombination in a small subset of the heterogeneous cell population. In this case, the DNA recombination may not be detectable by PCR with bulk cells. To obtain cells with a homogenous genetic background, we generated cell colonies from single cells. For 2 cscRNAs, the cDNA and genomic DNA from each of these single-cell originated colonies were then subjected to PCR amplification. As shown in Fig. 3c, only the PCR reactions with cDNA were positive, and the genomic DNA-based PCR were all negative. Therefore, as a proof of the concept, these 2 cscRNA sequences validated by RT-PCR in each of these 5 cell colonies, which supposedly has the same genetic background, should not be resulted from DNA recombination.

In addition, considering the potential artifacts during RT-PCR, we also used RNA fluorescence in situ hybridization (RNA FISH), a non-RT based assay, to directly probe the cscRNAs in the cells (Fig. 3d). Among 7 of the cscRNAs validated by RT-PCR, 3 of them (cscR-8-21, cscR-8-20, and cscR-2-22) were detected by RNA FISH. Importantly, the fluorescence signals were dramatically reduced upon knockdown of the cscRNAs but not upon knockdown of the host genes (Fig. 3d), which confirmed the specificities of the FISH probes. Note that the siRNAs targeting the cscRNAs did not have strong off-target effects on the parental transcripts (Fig. S9), whereas the siRNAs targeting the host genes all showed at least 80% knockdown efficiency.

**Fig. 4:**
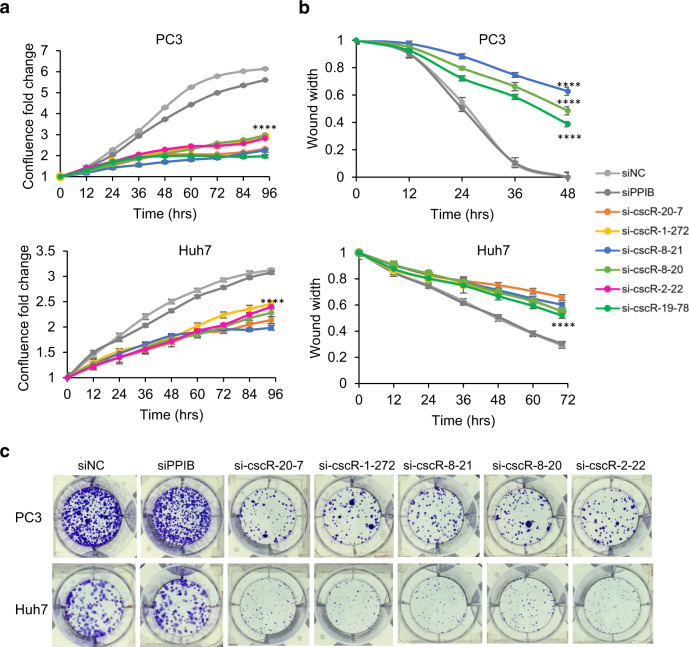
Biological relevance of the cscRNAs in cancer cells. **a** Cell proliferation curves of PC3 and Huh7 cells upon siRNA-mediated knockdown of the cscRNAs. The *y*-axis shows the cell confluence fold-change in relative to the confluence at time 0. The error bars represent the ±SD of three biological replicates. ****p = 0.0001 from two-sided *t*-test. Source data are provided as a Source data file. **b** Wound-healing assays showing the cell migration rates of PC3 and Huh7 upon siRNA-mediated knockdown of the cscRNAs. The *y*-axis shows the wound width at different time points in relative to the wound width at time 0. The error bars represent the ±SD of three biological replicates. ****p = 0.0001 from two-sided *t*-test. The representative images of the wounds are shown in Fig. S10. Source data are provided as a Source data file. **c** Formation of PC3 and Huh7 cell colonies upon siRNA-mediated knockdown of the cscRNAs.

Finally, to explore the potential biological relevance of the cscRNAs, we focused on the experimentally validated cscRNAs and simply tested the effects of cscRNA knockdown in cancer cells. Knockdown of 6 cscRNAs (cscR-20-7, cscR-1-272, cscR-8-21, cscR-8-20, cscR-2-22, and cscR-19-78) (Fig. S9a), which did not affect the levels of the parental transcripts (Fig. S9b), significantly repressed proliferation, colony formation and/or migration of the PC3 and Huh7 cells (Figs. 4a–c, S10), suggesting that these cscRNAs are of potentially high relevance in the cancer cells. On the other hand, knockdown of other 8 cscRNAs did not result in any phenotypic responses of the PC3 and Huh7 cells, including cell proliferation, migration, and colony formation. This, however, does not necessarily mean that these cscRNAs are biologically inert. More and different types of the assays would be needed to fully elucidate their functions.

### Origination of cscRNAs mainly from convergent transcription

**Fig. 5:**
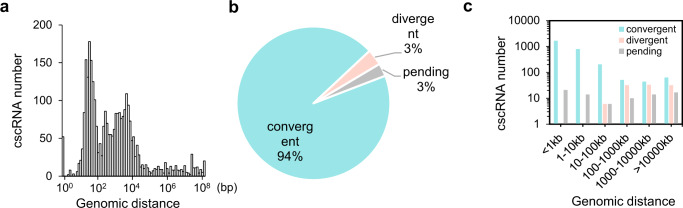
Convergent transcription potentially giving rise to the cscRNAs. **a** Distribution of the genomic distances between each pair of the cross-strand junction sites of the recurrent cscRNAs. **b** Proportions of the recurrent cscRNAs that were originated from convergent transcription and divergent transcription, and the cscRNAs of which the originations were undetermined. **c** The cscRNAs were first partitioned into different groups based on the genomic distances between the cross-strand junction sites. Within each group, the proportions of the recurrent cscRNAs in the 3 categories introduced above were shown on the *Y* axis.

As introduced above, for a cscRNA, the 3′ end of the upstream RNA fragment (5′ fragment) was ligated to the 5′ end of the downstream RNA fragment (3′ fragment) (Fig. 1a). These two junction sites, i.e., the 5′ and the 3′ junction sites, by definition, are mapped back to the two template DNA strands. Interestingly, for most of the cscRNAs, the two paired junction sites are overwhelmingly close on the genome (Fig. 5a). More than 1/3 (37.73%, 1137/3013) of the recurrent cscRNAs have their 5′ and 3′ junction sites within 100 bp on the genome DNA, over half (55.95%, 1686/3013) of the cscRNAs within 1000 bp, and 83% (2501/3013) within 10,000 bp (Fig. 5a), which is the average size of human genes.

Therefore, the two RNA fragments forming a cscRNA are transcription products of the opposite DNA strands within a very small region of the genome. Such bi-directional transcriptions fall in two categories, convergent and divergent transcriptions. Interestingly, vast majority of the cscRNAs are products of convergent transcription (Fig. 5b), where head-to-head transcription machineries move toward each other. Only a very small fraction of the cscRNAs can be attributed to divergent transcription (Fig. 5b, c), and the 5′ and 3′ junction sites of these cscRNAs tend to be far away from each other on the genome (Fig. 5c).

**Fig. 6:**
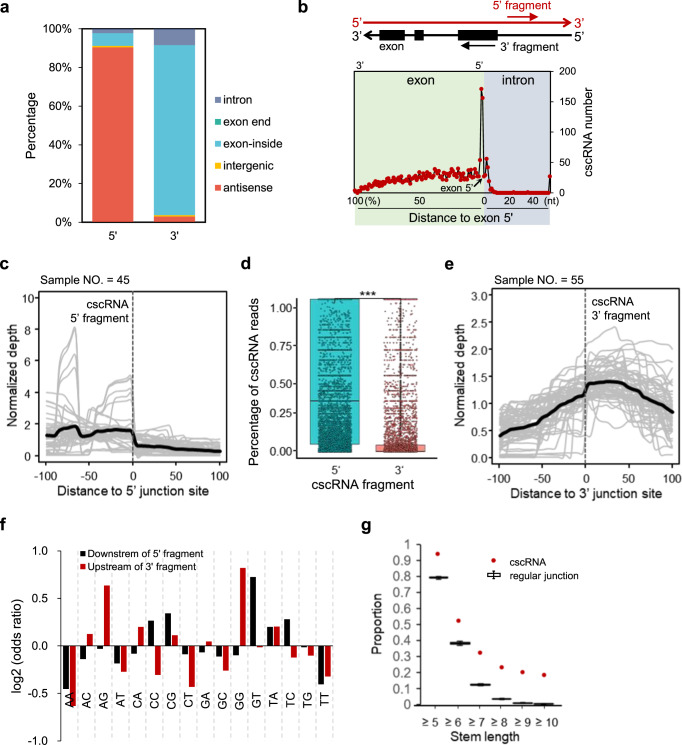
Features of the cross-strand junctions related to cscRNA biogenesis. **a** Proportions of the cscRNAs categorized according to the genomic annotations of the 5′ or 3′ junction sites. antisense: the antisense strand of an annotated gene; intergenic: intergenic region of the genome not being annotated to any gene; exon-inside: inside of an exon region; exon end: the 5′ cross-strand junction site being the 3′ end of an exon or the 3′ junction end being the 5′ end of an exon; intron: inside of an annotated intron. **b** Distributions of the distances between the 3′ junction sites and the closest exon 5′ ends. Note that for the 3′ junction sites located inside of an exon (left part of the plot), their distances to the exon 5′ ends were transformed into percentages via normalization by the length of the hosting exons. For the junction sites located in the introns, the distances (nt) to the closest downstream exon 5′ ends were recorded (right part). **c** Average read depths along the genome around the 5′ junction sites (200 nt in total). For each sample (represented by a gray line), the value of each position was calculated by taking the average of the read depths of the multiple cscRNAs expressed in the particular sample. All the values along the 200-nt region were then normalized by their average, so that the different lines, indicating different samples, are in the same normalized scale. To control the stochastic noise, we only used the 5′ junction sites around which the average read depths within the 200-nt region were not lower than 5. Such analyses were performed for 45 samples, which have at least 5 cscRNAs with the 5′ junction sites passing the filter above. Finally, the average of the 45 samples on each position was shown as the thick black line. **d** The RNA-seq reads covering each of the 5′ or 3′ cross-strand junction sites in each sample are composed of two types of the reads, i.e., the reads that supported the cscRNAs and the reads that were mapped to the genome and therefore supporting the regular RNA transcripts. The proportions of the RNA-seq reads that supported the cscRNAs were summarized as box plots for the 5′ and 3′ junction sites of all the recurrent cscRNAs (Two-sided *t*-test, *p*-value = 2.2e−16). The median value is shown as the line and the average as the cross. **e** Average read depths along the genome around the 3′ junction sites (200 nt in total). The lines were prepared with the same method in panel d, for 55 samples, which have at least 5 cscRNAs with the 3′ junction sites passing the filter. Finally, the average of the 55 samples on each position was shown as the thick black line. **f** Odds ratios of the dinucleotide motifs at the downstream of the 5′ junction sites and the upstream of the 3′ junction sites of the recurrent cscRNAs. 10,000 randomly picked positions of the genome was used as the background. **g** The percentages of the recurrent cscRNAs that have complementary sequences between their 5′ and 3′ fragments. As a reference, the probabilities of detecting complementary sequences between the up- and downstream regions of 10,000 randomly selected exon–exon junction sites were counted. Such randomization was performed for 100 times, and the 100 probabilities were summarized as box plots (Two-sided *t*-test *P*-value from left to right: 1.08E−73, 1.11E−61, 7.76E−92, 2.02E−115, 6.92E−120, 6.17E−130). The median value is shown as the line and the average as the cross. Source data are provided as a Source data file.

Furthermore, as shown in Fig. 6a, most of the 3′ junction sites of the cscRNAs are located in the exons of the annotated genes, whereas the 5′ junction sites mostly fall in the anti-sense regions of the annotated genes. Interestingly, while the majority of the 3′ junction sites are located inside of the exons or introns, they are highly enriched near the exon ends (Fig. 6b). The similar pattern is observed in mouse as well (Fig. S11). Note that only a small percentage (3.9%) of the cscRNAs were formed by fusions between two previously annotated transcripts from the different strands. Most of these cross-strand junctions were between the annotated exons.

Next, around the 5’ and 3’ junction sites of the cscRNAs, we looked into the approximal percentage of the two transcripts from convergent transcription that take part in the cross-strand fusions. For the purpose of comparison, we randomly selected the antisense regions and the 5′ exon ends as background of the 5′ and 3′ cross-strand junction sites, respectively.

First of all, taking the 45 samples with the highest numbers of the cscRNAs, for the 5′ transcripts, i.e., the transcripts that contributed the 5′ fragment of the cscRNAs, the average RNA read depth showed a sharp drop at the cross-strand junction sites (Fig. 6c). By contrast, for the normal antisense regions, the read densities keep consistent around the junction sites, which is well expected (Fig. S12a). Indeed, for the cscRNAs in a specific sample, at the 5′ junction sites, the RNA-seq reads originated from the cscRNAs could take major proportions of the total reads, which is composed of the cscRNAs reads and the reads of the other regular transcripts (Fig. 6d). These results above suggest that for many of the 5′ transcripts generating the cscRNAs, their predominant destinies are fusion with the RNA transcripts from the opposite direction.

However, this is not the case for the 3′ transcripts of the cscRNAs. At the 3′ junction sites, the RNA-seq reads originated from the cscRNAs only account for small proportions of all the reads combined (Fig. 6d). The average read depths around the 3′ junction sites did not change dramatically (Fig. 6e), unlike the sharp drop around the 5′ junction sites (Fig. 6c). As expected, there is a sharp ramp around the normal intron–exon junction sites (Fig. S12b). Therefore, unlike the 5′ transcripts, only small proportions of the 3′ transcripts took parts in the cross-strand RNA fusion events to form the cscRNAs. Together, these special read density patterns around the 5′ and 3′ junction sites of the cscRNAs suggest potentially non-canonical transcriptional and post-transcriptional processing of the precursor RNAs during cscRNA biogenesis.

### cscRNAs are potentially related to non-canonical RNA splicing

All the features above of the cscRNAs prompted us to explore whether such cross-strand fusion events are potentially related to some non-canonical RNA splicing. The process of RNA splicing generates splicing donor and acceptor sites. For the cscRNAs, the canonical splicing donor site “GT” is relatively enriched in the downstream genome sequence of the 5′ junction sites, whereas the canonical acceptor site “AG” is enriched in the upstream sequence of the 3′ junction sites (Fig. 6f). However, such enrichments are too weak to conclude that the canonical RNA splicing takes place during formation of the cscRNAs. In fact, studies of other types of the chimeric RNAs also showed that only a small fraction of the non-canonical fusion events have the canonical splicing donor and acceptor sites^28^. Finally, it is worth noting that most of the 3′ junction sites, if not located at the 5′ exon ends, are located inside the exons or introns with a strong enrichment near the exon ends (Fig. 6b). This suggests that the origination of cscRNA is potentially related to RNA splicing, especially for the 3′ fragments of the cscRNAs.

As introduced in the previous section, the cscRNAs are mostly originated from focal convergent transcription, and their junction sites are usually very close on the genome (Fig. 5a). In other words, the two RNA fragments being fused to form the cscRNAs are geometrically close with each other after being transcribed. This lays the geometrical basis for the formation of the cscRNAs. Furthermore, for the cscRNAs of which the junction sites are within a short distance in the genome, e.g., within few hundreds of bp, the two RNA fragments by definition should share a long range of sequences that are reverse complimentary to each other. This could further promote the closeness of the two RNA fragments and formation of the cscRNAs. For the cscRNAs that are not so close on the genome, e.g., junction sites >1000 bp away, it is also very interesting that the two RNA fragments are more frequently found to share some reverse complimentary sequences than other randomly selected exon sequences (Fig. 6g). These complementary sequences in the two RNA fragments may have also promoted their spatial proximity, which could be critical for the formation of the cscRNAs. Finally, we looked for the actual RNA-seq reads that directly support such complementary sequences in the downstream regions of the cross-strand junction sites. For 32% of the cscRNAs, complementary RNA-seq reads were found to be mapped to the downstream regions of the junction sites on the genome. This supports the hypothesis that the overlapping and complementary regions in the precursor RNAs may have helped generation of the cscRNAs, which is similar to the potential mechanism for biogenesis of the circRNAs^27,29^. More thorough studies are certainly needed to elucidate the clear machinery in the follow-up studies.

## Discussion

Gene fusion due to DNA rearrangement has been frequently shown in genetic disorders such as cancer^12,13,48^. More recently, chimeric RNA species as results of post-transcriptional RNA processing have been emerging and exhibiting physiological functions in disease and normal processes^10,21,49^. These gene fusion events taking places at the RNA level generally fall in two categories, i.e., trans-splicing between transcripts from different genomic loci and transcription read-through between adjacent genes^17,50–53^. It has been well-recognized that the trans-splicing events, although taking places between distal genes, depend on spatial proximity between the genes in the 3-D structure of the chromosome^30^.

Convergent transcription of the two DNA strands is prevalent in the genomes of various organisms^54–58^, which by definition generates pairs of transcripts that are geometrically close before leaving their transcription sites. We made a hypothesis that such transcripts, potentially upon post-transcription processing such as splicing, partial degradation, and endo- or exo-nuclease digestion, could be subjected to RNA fusion and therefore give rise to cscRNAs as a different type of chimeric RNA species. Based on the RNA deep-sequencing data from the ENCODE consortium and other sources, we designed a specialized bioinformatics pipeline cscMap to search for the RNA fusion events between any two precursor RNAs transcribed from the opposite strands of DNA as templates. To our surprises, cscRNAs are frequently found in various types of human and non-human samples and in both cancerous and normal cell contexts. Furthermore, such cross-strand RNA fusions exhibited extensive tissue-specificity.

By taking advantage of the paired-end sequencing strategy and only using the normally un-mappable reads, the pipeline of cscMap ensures that the cross-strand RNA junction sites are perfectly covered by sequencing reads as direct evidence and the events of strand-shifting have further supports from the paired reads. However, the canonical library preparation for total RNA-seq involves reverse transcription and sequencing of the PCR-amplified cDNA molecules. As mentioned previously, this raised the concern of 5′ ligation or 3′ self-priming during reverse transcription, which could potentially introduce artificial cscRNAs. As most of the cscRNAs are composed of the 3′ fragments from annotated mRNA transcripts and the 5′ fragments from the anti-sense transcripts (Fig. 6a), the potential reverse transcription artifact, if any, would be likely due to the mechanism of 5′ ligation of the mRNA species. In this scenario, the 5′ junction sites of the cscRNAs on the antisense strand should be located toward the downstream of the 3′ junction site on the mRNA strand (Fig. 1f). However, our analysis did not show such a pattern of the 5′ junction sites being enriched in the downstream of the 3′ junction sites (Fig. S13). In fact, the 5′ junction sites are slightly more enriched in the upstream of the 3′ junction sites. This observation strongly argues against the possibility of the cscRNAs being just artifacts due to 5′ ligation during reverse transcription.

The 3′ junction sites are highly enriched around, but not exactly located at, the annotated 5′ exon ends (Fig. 6b). In addition, the canonical splicing donor (GT) and acceptor (AG) sites are enriched, but only weakly, around the junction sites. These observations suggest that the cross-strand RNA fusion events, especially for the 3′ junction sites, are potentially related to RNA splicing, but not simply controlled by the canonical machinery of RNA splicing. The cross-strand RNA fusion events predominantly take place between the RNA transcripts from local convergent transcription (Fig. 5a–c). It has been frequently shown that RNA polymerase II (RNAPII) molecules collide with each other during convergent transcription, resulting in arrest of elongation in one or both directions^59–61^. Therefore, it is a plausible hypothesis that the 5′ RNA fragments of the cscRNAs were generated by the restricted convergent transcription due to transcription collision, whereas the 3′ fragments, as discussed above, were related to a type of non-canonical RNA splicing. In general, the highly expressed genes have higher chances to produce cscRNAs (Fig. S14). This is expected as the genomic regions undergoing active transcription would be more likely to host the non-canonical RNA processing events as well. However, it is also worth noting that some of the parent genes of the cscRNAs are not abundantly expressed. The machinery of cscRNA biogenesis and its potential impact on the parent gene is certainly worth further investigations.

As the first systematic survey of the non-canonical fusion between the sense and anti-sense transcripts, the current study has been focused on de novo identification and characterization of the cscRNAs in different biological samples of human and multiple other species. Although fully illustrating the biological functions of the cscRNAs is out of scope of the current study, we are convinced that the cscRNAs are unlikely to be simply transcription noise because of the following reasons. (1) Even with the highly conservative settings and multiple stringent filters implemented by the pipeline of cscMap, most of the 3013 recurrent cscRNAs were still identified in at least two different types of the biological samples (Fig. 2c). Many of the cscRNAs were repeatedly found in more than 10 samples. In other words, these cross-strand junctions at exactly the same sites take place in multiple independent samples. This is unlikely to be just results of transcription noise. (2) The cross-strand junction events show a series of special features (Figs. 5, 6), including the short distance between the 5′ and 3′ junction sites on the genome, the enrichment near the exon ends, enriched motifs around the junction sites. (3) The abundances of the cscRNAs are in general not trivial. The highly expressed cscRNAs are comparable to some annotated mRNAs and highly expressed long non-coding RNAs (Fig. 2a, b), suggesting that they are not just transcription noise or some sort of junk RNA to be recycled. (4) The cscRNAs showed high context dependency. However, compared to the biologically distinct samples, the samples from similar types of tissues or primary cells do share more common cscRNAs (Fig. 2d). Such expression patterns suggest context-dependent relevance or functions of the cscRNAs in different types of tissues. (5) Finally, as discussed above, despite being still preliminary, the function assays upon cscRNA knockdown in Fig. 4 imply potential biological relevance of the cscRNAs.

In summary, from the systematic survey presented in the manuscript, we show that the non-canonical fusion between the sense and anti-sense transcripts is prevalent in human and multiple other species. We have characterized the basic features related to biogenesis of the cscRNAs, such as their abundances, experimental validations, annotations of the cross-strand junction sites, and other sequence features around the non-canonical junction sites. Next, the biological functions of the cscRNAs, especially their biological relevance in disease and normal physiology, which could be highly complicated and specific to each individual cscRNA, would certainly worth further in-depth investigations. Therefore, we believe that the current study should serve as an insightful resource to inspire and support further elucidation of this additional layer of the transcriptome complexity.

## Methods

### Data sources

The details of all the datasets used in the present study are provided in Supplementary Data 1. Most of the rRNA-depleted total RNA-seq datasets were obtained from the ENCODE project. They are all paired-end sequencing reads with read length 101 bp. The dataset covers 271 human samples (24 immortalized cancer cell lines, 52 primary cells, and 39 non-cancerous tissues, each of which has at least two biological replicates) and 20 mouse samples (2 cancer cell lines, 1 stem cell, 5 tissues, each of which has at least two biological replicates).

The datasets from other species were obtained from the GEO database (Supplementary Data 1), including *zebrafish* (GSE73615 and GSE74929), *yeast* (GSE110413 and GSE99170), *C. elegans* (GSE79375 and GSE79375), *fruit fly* (GSE83877 and GSE101603), and *E. coli* (GSE41190). Datasets from some human samples were also downloaded from the GEO database, including MCF7 cells (GSE94372 and GSE89888), PC3 cells (GSE65112 and GSE48230), and lung tissues (GSE60052 and GSE52248).

The whole-genome DNA-seq data were obtained from the GEO database (GSE48216). The dataset includes 9 breast epithelial cell lines (MCF10A, MCF10F, MCF12A, HCC1143, HCC1143BL, HCC1187, T4, T47D, T47D_KBluc).

### Design of cscMap for identification of the cscRNAs

The pipeline of cscMap for identification of the cscRNAs is shown in Fig. S1. The reference genome of human (hg19) and mouse (mm10) were downloaded from the UCSC Genome Browser, and the reference genomes of other species were from the Ensemble Genome Browser (*zebrafish*: GRCz11, *C. elegans*: WBcel235, *fruit fly*: BDGP6, *Saccharomyces*: R64-1-1, *E. coli*: Escherichia_coliK-12_substr.MG1655). The genome annotation files of both human and mouse were obtained from GENCODE (human: v19, mouse: vM17), and the annotation files of all the other species were from the Ensemble Genome Browser.

Paired-end reads were first mapped to the reference genome by TopHat 2.0^62^. The program was run with the parameters “-m 2” to control the mismatches no more than 2, “-G genome_annotation.gtf” to use the transcriptome as the reference, and “--library-type fr-firststrand” for strand-specific alignments. All the other default parameters were used. The unmapped reads were then used as inputs of the following steps of cscMap.

Bowtie 2.0 in its local mode (parameter “--local”)^63^ was then used for mapping of the normally unmapped RNA-seq reads from above to two different locations. Specifically, the reads were mapped twice at the same time by using the parameters “--norc” and “--nofw”, which allow the program to seek alignments of the reads to the sense and antisense strands separately. This identifies the reads, of which the two fragments were mapped to the two opposite strands on the same chromosome. The minimal length of the read fragment mapped to either of the two DNA strands was set to be 20 nt. The two nucleotides between which the read was truncated into two fragments were defined as the 5′ and 3′ cross-strand junction sites. Finally, when one end of the paired-end reads was truncated into two fragments and consequently mapped to the two DNA strands, the other end (the mate read) was mapped to the upstream of 5’ junction site or the downstream of 3′ junction site, depending on the mapping scenario of the first end that were truncated and mapped.

The candidate cscRNAs were then subjected to multiple layers of filters: (1) The cscRNAs of which the sequences around the cross-strand junction sites (±20 nt) overlap with the repeat sequences (obtained from the repeat database Repbase^64^) by at least 1 nt were removed. (2) The junction sequences were again aligned to the genome by Blast, and the cscRNAs showing high similarity (identity >80%, alignment length >90 nt) to the genome were removed. (3) The cscRNAs showing features of 3′ self-priming or 5′ ligation were removed. Specifically, as shown in Fig. 1e, f, a stem-loop structure formed by the upstream sequence of the 5′ cross-strand junction site indicates 3′ self-priming, and a stem-loop structure at downstream of the 3′ junction site indicates 5′ ligation. Here base-pairing for at least 4 bp were defined as the stem. The loop length, i.e., distance between the base-pairing sequences, was limited between 0 and 100 nt.

Supplementary Data 2 provides the library of all the cscRNA identified in the human samples from ENCODE. The information of cscRNAs includes the genomic locations of the 5′ and 3′ junction sites, the genomic annotations of the 5′ and 3′ fragments, the RNA-seq read counts, the biological samples, and the cscRNA sequences recovered by the RNA-seq reads.

### Alignment of the cscRNA sequences with the DNA-seq reads and the mouse cscRNAs

To capture the cscRNAs that are potentially resulted from DNA rearrangement, we collected 9 DNA-seq datasets (GSE48216, breast epithelial cell lines: MCF10A, MCF10F, MCF12A, HCC1143, HCC1143BL, HCC1187, T4, T47D, T47D_KBluc) for alignments between the DNA sequences and the cscRNA sequences around their cross-strand junction sites (at least 20 nt in up- and downstream of the junction sites). Blast software was used, with the parameters “-subject cscRNA_sequence.fa” to set the cscRNA sequences as reference, “-query DNA_sequence.fa” to align the DNA sequences on the cscRNA reference, and “-outfmt” to customize the output contents. The cscRNAs, of which the RNA fusion events were supported by DNA-seq reads, are marked in Supplementary Data 2.

Similarly, the human cscRNA sequences around their cross-strand junction sites (20 nt in up- and downstream of the junction sites) were aligned with the mouse cscRNA sequences. Blast was used with the default parameters. In addition, the 5’ and the 3’ junction sites were also compared between human and mouse cscRNAs. Specifically, with the Blast software, the 5′ fragments (20 nt in upstream of the junction sites) or the 3′ fragments (20 nt in downstream of the junction sites) were aligned to the 5′ fragment (25 nt in upstream of the junction sites) or 3′ fragments (25 nt in downstream of the junction sites) of the mouse cscRNAs, respectively. In addition to the sequence similarity, the 5′ or 3′ junction sites were required to be perfectly aligned between human and mouse cscRNAs. The conserved cscRNA fragments (3′ or 5′), or the full-length are marked in Supplementary Data 2.

### Quantifications of the cscRNA abundances

Fragments per kilobase of transcript per million mapped reads (FPKM) or the raw read counts of the cscRNAs were used as quantifications of the cscRNA abundances. To calculate the FPKM, the length of a given cscRNA was estimated by the spanning region of RNA-seq reads that covered the cross-strand junction sites.

### Cell culture

Cell lines were purchased from American Type Culture Collection (ATCC) and cultured in a humidified incubator with 5% CO_2_ at 37 °C. HUH7 and A549 cells were cultured in Dulbecco’s modified Eagle medium (High glucose, BI). PC3 cells were cultured in Roswell Park Memorial Institute 1640 (RPMI-1640, BI). All the media were supplemented with 10% fetal bovine serum (BI).

### RNA extraction and real-time qPCR analysis

For validations of the cscRNAs with PCR, total RNA was extracted and purified with TRIzol (Invitrogen) from the cells by following the manufacturer’s instruction. 2 μg of the purified RNA was subjected to DNase I treatment (Invitrogen), followed by reverse transcription with the High-Capacity cDNA Reverse Transcription Kit (Invitrogen). The cDNA was diluted 10 times in nuclease-free water and used as the templates for PCR amplification. On the other hand, the genomic DNA was extracted with the AxyPrepTM Multisource Genomic DNA Miniprep Kit (Axygen) from the cells. 200 ng gDNA in nuclease-free water was used as the template for PCR amplification.

The PCR amplifications were carried out on a thermal cycler with the 1× RealStar Green Power Mixture (GenStar) and 400 nM for each of the forward and reverse primers. The primer sequences are provided in Supplementary Table 1.

The PCR products were visualized by electrophoresis in 1% agarose gel with Gel Safe DNA stain (Invitrogen). To obtain the DNA sequences, the PCR products were purified with the Gel Extraction Kit (Vazyme) and then subcloned into the pGEM-T vector (Promega). The plasmids were transformed into DH5α competent cells (Vazyme), which were then spread on an AMP resistance screening LB agar plate (containing X-gal and IPTG). The plates were incubated at 37 °C overnight, and the recombinant cells in the white colonies were collected for Sanger sequencing.

In addition to the PCR assays with bulk cells, the similar assays were also performed with cell colonies originated from single cells. Specifically, the cells were grown in dishes and resuspended after trypsinization. The cells were diluted and seeded in 48-well plates. The wells were checked under the microscope, and only the ones with just one cell were used for growing of the cell colonies. After 2–3 weeks, the colonies from single cells were harvested. The RNA and DNA were extracted and purified for the PCR assays as introduced above.

### RNA fluorescence in situ hybridization (FISH) and immunofluorescence microscopy

The RNA-FISH assay was performed as previously described^65,66^. Specifically, about 0.15 kb fragments covering the cscRNA junction sites were amplified using the primer sets (cscR-8-21: GTCTAAAGCTTCGGCACAAGGG and AGGCCTTACCATCTTCTTGGTT, cscR-8-20: TCGTGGCCTGGTCTCCATTATTT and CTCCAGGCCTTACCATCTTCTTG, cscR-2-22: GTCGCAGCAACAACTTCCAG and ACACTAACCACATACTCCACTGT). The fragments were then cloned into pGEM-T (Promega). The cscRNAs probes were synthesized with DIG RNA labeling Mix (Roche) and SP6 or T7 polymerase (Thermo), according to the manufacturers’ instructions.

Cells were seeded onto a Nunc glass bottom dish, washed with PBS solution, fixed with 4% paraformaldehyde solution for 10 min and then washed with 1× PBS solution for three times at room temperature (RT). Subsequently, the fixed cells were permeabilized with 0.5% Triton X-100 in PBS solution for 5 min and then rinsed with PBS solution. The slides were incubated with prehybridization solution (2× SSC, 1× Denhardt solution, 50% (vol/vol) formamide, 10 mM EDTA (pH 8.0), 100 μg/mL yeast tRNA, and 0.01% Tween-20) at 55 °C for 2 h. The prehybridized slides were then incubated with hybridization solution (prehybridization solution containing 5% (wt/vol) dextran sulfate and 2 μg/mL digoxigein (DIG)-labeled RNA probe at 55 °C for 16 h. After hybridization, the slides were washed twice with prewarmed wash buffer (2× SSC, 50% (vol/vol) formamide, and 0.01% Tween-20) at 55 °C for 30 min, and then excess RNA probes were digested by incubating with 10 μg/mL RNase A in NTET buffer (10 mM Tris·HCl (pH 8.0), 1 mM EDTA, 500 mM NaCl, and 0.1% Tween-20) at 37 °C for 1 h. The slides were then washed once with buffer A (2× SSC and 0.01% Tween-20) at 55 °C for 30 min and twice with buffer B (0.1× SSC and 0.01% Tween-20) at 55 °C for 30 min. For detection, the slides were washed with Tris buffered saline solution containing 0.1% Tween-20 (TBST), incubated with 3% (wt/vol) BSA blocking solution in TBST at RT for 1 h, and then incubated with sheep anti-DIG antibodies (Roche, 11333089001) diluted (1000 X) in blocking solution at 4 °C overnight. Unbound antibodies were removed by three 15-min washes in TBST. The slides were then incubated with a Donkey Anti-Sheep IgG H&L (Alexa Fluor® 594) (Abcam, ab150180) fluorophore-conjugated secondary antibody diluted (1000 X) in blocking solution for 1 h at RT. After washing, the slides were mounted with medium containing DAPI (ZSBIO, ZLI-9557). Fluorescent images were visualized at RT under a microscope (Zeiss LSM980 Airyscan2) equipped with Plan Apochromat ×60/1.4 objective lenses (Zeiss). Imaris 9.6 software (Bitplane) was used for image acquisition and processing. All overlaid images were transferred as high-resolution TIFF files.

### Small interfering RNA (siRNA) synthesis and transfection

The siRNAs specifically targeting cscRNAs, PPIB, and non-targeting control siRNA were synthesized by GenePharma (Supplementary Table 2). siNC and siPPIB served as negative controls. Cells were transfected with the siRNAs using Lipofectamine RNAiMAX Reagent (Invitrogen), respectively, following the manufacturer’s protocol.

### Cell proliferation, colony formation assays, and wound-healing assays

After different types of gene perturbations, the cells were cultured in 96-well plates, at the starting density of 10,000 cells per well. The IncuCyte live-cell imaging and analysis system (ESSEN Bioscience) was used to monitor real-time cell proliferation and morphology changes. Cell proliferation was quantified by measuring the occupied area (% confluence) in the cell images over time. The proliferation curves were made with confluence fold change (FC) at different time points in relative to the confluence at time 0.

For colony formation assays, 1000 cells were seeded in the 6-well plates and incubated with normal growth medium for 14 days. Then cells were fixed and stained with 0.5% crystal violet for 15 min. Lastly, the colonies were imaged via a camera or a microscope.

The wound healing assay was performed to monitor and quantify cell motility. Briefly, cells were seeded in a 96-well plate at 30,000 cells per well and allowed to reach confluence before the surface was uniformly scratched across the center of the well by an Essen wound maker (Essen Bioscience). The wells were then rinsed with fresh medium to remove floating cells, and the wound healing process was monitored continuously in the IncuCyte live-cell imaging system (Essen Bioscience). Images were obtained at each set time point and then analyzed by the IncuCyte scratch wound assay software to quantify wound healing. Data were expressed as wound widths.

### Reporting summary

Further information on research design is available in the Nature Research Reporting Summary linked to this article.

## Supplementary information

Supplementary Figures and Tables

Dataset 1

Dataset 2

Dataset 3

Description of additional supplementary files

Supplementary Software

Reporting Summary

## Data Availability

The data supporting the findings of this study are available from the corresponding authors upon reasonable request. Most of the human rRNA-depleted total RNA-seq datasets were obtained from the ENCODE project. The datasets from other species were obtained from the GEO database. The details of all the datasets used in the present study are provided in Supplementary Data 1. The cscRNA species identified in this study are provided in Supplementary Data 2 and 3. Source data are provided with this paper.

## Source data

Source data File

## Code availability

The cscMap pipeline has been provided as a Supplementary software and deposited in github (https://github.com/xryanglab/cscMap).

## References

[1] Mercer TR (2011). Targeted RNA sequencing reveals the deep complexity of the human transcriptome. Nat. Biotechnol..

[2] Brown JB (2014). Diversity and dynamics of the *Drosophila* transcriptome. Nature.

[3] Barbosa-Morais NL (2012). The evolutionary landscape of alternative splicing in vertebrate species. Science.

[4] Merkin J (2012). Evolutionary dynamics of gene and isoform regulation in Mammalian tissues. Science.

[5] Gupta SK, Luo L, Yen L (2018). RNA-mediated gene fusion in mammalian cells. Proc. Natl Acad. Sci. USA.

[6] Heim S, Mitelman F (2008). Molecular screening for new fusion genes in cancer. Nat. Genet.

[7] Dehghannasiri R (2019). Improved detection of gene fusions by applying statistical methods reveals oncogenic RNA cancer drivers. Proc Natl Acad Sci USA.

[8] Frenkel-Morgenstern M (2015). ChiTaRS 2.1–an improved database of the chimeric transcripts and RNA-seq data with novel sense-antisense chimeric RNA transcripts. Nucleic Acids Res..

[9] Hanahan D, Weinberg RA (2011). Hallmarks of cancer: the next generation. Cell.

[10] Li H (2008). A neoplastic gene fusion mimics trans-splicing of RNAs in normal human cells. Science.

[11] Jividen K, Li H (2014). Chimeric RNAs generated by intergenic splicing in normal and cancer cells. Genes Chromosomes Cancer.

[12] Rowley JD (1973). Letter: A new consistent chromosomal abnormality in chronic myelogenous leukaemia identified by quinacrine fluorescence and Giemsa staining. Nature.

[13] Shaw AT (2011). Effect of crizotinib on overall survival in patients with advanced non-small-cell lung cancer harbouring ALK gene rearrangement: a retrospective analysis. Lancet Oncol..

[14] Rabbitts TH (1994). Chromosomal translocations in human cancer. Nature.

[15] Rowley JD (1999). The role of chromosome translocations in leukemogenesis. Semin Hematol..

[16] Gingeras TR (2009). Implications of chimaeric non-co-linear transcripts. Nature.

[17] Zhang Y (2012). Chimeric transcript generated by cis-splicing of adjacent genes regulates prostate cancer cell proliferation. Cancer Disco..

[18] Lai J (2010). A variant of the KLK4 gene is expressed as a cis sense-antisense chimeric transcript in prostate cancer cells. RNA.

[19] Zhang C (2003). A candidate chimeric mammalian mRNA transcript is derived from distinct chromosomes and is associated with nonconsensus splice junction motifs. DNA Cell Biol..

[20] Velusamy T (2013). Recurrent reciprocal RNA chimera involving YPEL5 and PPP1CB in chronic lymphocytic leukemia. Proc. Natl Acad. Sci. USA.

[21] Yuan H (2013). A chimeric RNA characteristic of rhabdomyosarcoma in normal myogenesis process. Cancer Disco..

[22] Babiceanu M (2016). Recurrent chimeric fusion RNAs in non-cancer tissues and cells. Nucleic Acids Res..

[23] Frenkel-Morgenstern M (2012). Chimeras taking shape: potential functions of proteins encoded by chimeric RNA transcripts. Genome Res..

[24] Rickman DS (2009). SLC45A3-ELK4 is a novel and frequent erythroblast transformation-specific fusion transcript in prostate cancer. Cancer Res..

[25] Chwalenia K (2019). A cell-based splicing reporter system to identify regulators of cis-splicing between adjacent genes. Nucleic Acids Res.

[26] Koller U (2015). Trans-splicing improvement by the combined application of antisense strategies. Int J. Mol. Sci..

[27] Ivanov A (2015). Analysis of intron sequences reveals hallmarks of circular RNA biogenesis in animals. Cell Rep..

[28] Li X (2009). Short homologous sequences are strongly associated with the generation of chimeric RNAs in eukaryotes. J. Mol. Evol..

[29] Zhang XO (2014). Complementary sequence-mediated exon circularization. Cell.

[30] Rodriguez-Martin B (2017). ChimPipe: accurate detection of fusion genes and transcription-induced chimeras from RNA-seq data. BMC Genomics.

[31] Benelli M (2012). Discovering chimeric transcripts in paired-end RNA-seq data by using EricScript. Bioinformatics.

[32] Abate F (2012). Bellerophontes: an RNA-Seq data analysis framework for chimeric transcripts discovery based on accurate fusion model. Bioinformatics.

[33] Jia W (2013). SOAPfuse: an algorithm for identifying fusion transcripts from paired-end RNA-Seq data. Genome Biol..

[34] Davidson NM, Majewski IJ, Oshlack A (2015). JAFFA: High sensitivity transcriptome-focused fusion gene detection. Genome Med.

[35] Frenkel-Morgenstern M (2013). ChiTaRS: a database of human, mouse and fruit fly chimeric transcripts and RNA-sequencing data. Nucleic Acids Res.

[36] Balamurali D (2019). ChiTaRS 5.0: the comprehensive database of chimeric transcripts matched with druggable fusions and 3D chromatin maps. Nucleic Acids Res.

[37] Gorohovski A (2017). ChiTaRS-3.1-the enhanced chimeric transcripts and RNA-seq database matched with protein-protein interactions. Nucleic Acids Res..

[38] Ransohoff JD, Wei Y, Khavari PA (2018). The functions and unique features of long intergenic non-coding RNA. Nat. Rev. Mol. Cell Biol..

[39] Derrien T (2012). The GENCODE v7 catalog of human long noncoding RNAs: analysis of their gene structure, evolution, and expression. Genome Res..

[40] Quinn JJ, Chang HY (2016). Unique features of long non-coding RNA biogenesis and function. Nat. Rev. Genet..

[41] Wang Y (2015). FusionCancer: a database of cancer fusion genes derived from RNA-seq data. Diagn. Pathol..

[42] Hu X (2018). TumorFusions: an integrative resource for cancer-associated transcript fusions. Nucleic Acids Res..

[43] Daemen A (2013). Modeling precision treatment of breast cancer. Genome Biol..

[44] Costello JC (2014). A community effort to assess and improve drug sensitivity prediction algorithms. Nat. Biotechnol..

[45] Lu Z, Matera AG (2014). Vicinal: a method for the determination of ncRNA ends using chimeric reads from RNA-seq experiments. Nucleic Acids Res..

[46] Houseley J, Tollervey D (2010). Apparent non-canonical trans-splicing is generated by reverse transcriptase in vitro. PLoS ONE.

[47] Frazee AC (2015). Polyester: simulating RNA-seq datasets with differential transcript expression. Bioinformatics.

[48] Downing JR (1995). Multiplex RT-PCR assay for the differential diagnosis of alveolar rhabdomyosarcoma and Ewing’s sarcoma. Am. J. Pathol..

[49] Jia Y, Xie Z, Li H (2016). Intergenically Spliced Chimeric RNAs in. Cancer Trends Cancer.

[50] Qin F (2016). Recurrent cis-SAGe chimeric RNA, D2HGDH-GAL3ST2, in prostate cancer. Cancer Lett..

[51] Ren S (2012). RNA-seq analysis of prostate cancer in the Chinese population identifies recurrent gene fusions, cancer-associated long noncoding RNAs and aberrant alternative splicings. Cell Res..

[52] Ren G (2014). Transcription-mediated chimeric RNAs in prostate cancer: time to revisit old hypothesis?. OMICS.

[53] Nacu S (2011). Deep RNA sequencing analysis of readthrough gene fusions in human prostate adenocarcinoma and reference samples. BMC Med. Genomics.

[54] Yelin R (2003). Widespread occurrence of antisense transcription in the human genome. Nat. Biotechnol..

[55] Katayama S (2005). Antisense transcription in the mammalian transcriptome. Science.

[56] Misra S (2002). Annotation of the Drosophila melanogaster euchromatic genome: a systematic review. Genome Biol..

[57] Dornenburg JE (2010). Widespread antisense transcription in *Escherichia coli*. mBio.

[58] Chatterjee A (2011). Convergent transcription confers a bistable switch in *Enterococcus faecalis* conjugation. Proc. Natl Acad. Sci. USA.

[59] Hobson DJ (2012). RNA polymerase II collision interrupts convergent transcription. Mol. Cell.

[60] Shearwin KE, Callen BP, Egan JB (2005). Transcriptional interference–a crash course. Trends Genet.

[61] Prescott EM, Proudfoot NJ (2002). Transcriptional collision between convergent genes in budding yeast. Proc. Natl Acad. Sci. USA.

[62] Kim D (2013). TopHat2: accurate alignment of transcriptomes in the presence of insertions, deletions and gene fusions. Genome Biol..

[63] Langmead B, Salzberg SL (2012). Fast gapped-read alignment with Bowtie 2. Nat. Methods.

[64] Bao W, Kojima KK, Kohany O (2015). Repbase update, a database of repetitive elements in eukaryotic genomes. Mob. DNA.

[65] Li X (2018). Oncogenic properties of NEAT1 in prostate cancer cells depend on the CDC5L-AGRN transcriptional regulation circuit. Cancer Res..

[66] Wang X (2021). Mutual dependency between lncRNA LETN and protein NPM1 in controlling the nucleolar structure and functions sustaining cell proliferation. Cell Res..

