# Supplementary material for: Identification of the cross-strand chimeric RNAs generated by fusions of bi-directional transcripts
Source: Nat Commun. 2021 Jul 30;12:4645. doi: 10.1038/s41467-021-24910-2 (PMC8324879; doi:10.1038/s41467-021-24910-2)
Supplement: Supplementary file 1 — Supplementary Figures and Tables [file 41467_2021_24910_MOESM1_ESM.pdf]

Supplementary Information

Identification of the cross-strand chimeric RNAs generated by fusions of bi-directional transcripts

Yuting Wang[1,2*], Qin Zou[1*], Fajin Li[1,2], Wenwei Zhao[1], Hui Xu[1], Wenhao Zhang[1], Haiteng Deng[1], Xuerui Yang[1]

[1] MOE Key Laboratory of Bioinformatics, Center for Synthetic & Systems Biology, School of Life Sciences, Tsinghua University, Beijing, China

[2] Joint Graduate Program of Peking-Tsinghua-National Institute of Biological Science, Beijing, China

*These authors contributed equally to this work.

Correspondence: Xuerui Yang, School of Life Sciences, Tsinghua University, Beijing 100084, China. Tel: 86-10-62783943. Email: yangxuerui@tsinghua.edu.cn

Figure S1

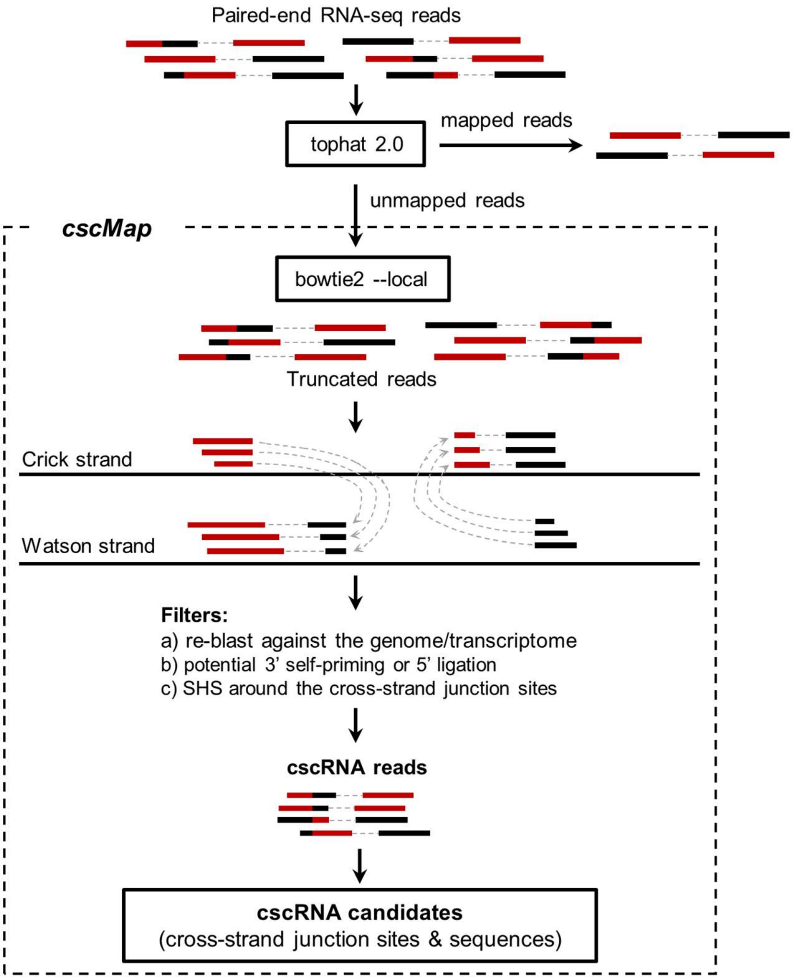

Figure S1. The pipeline of cscMap.

Schematic diagram showing the mapping strategies with PE RNA-seq reads for identification of the cscRNAs and the filtering criteria.

Figure S2

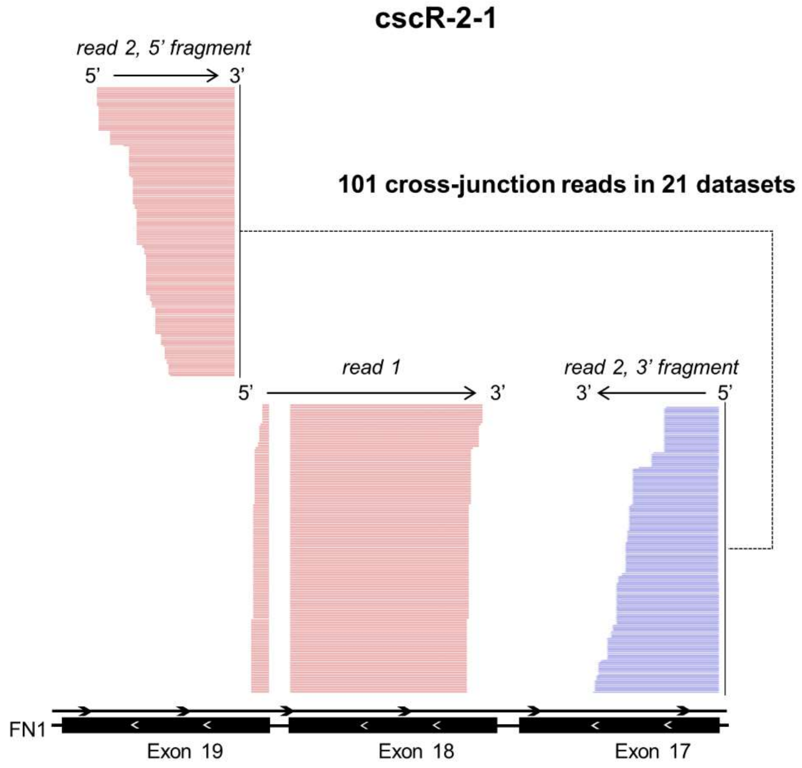

Figure S2. RNA-seq reads of cscR-2-1 in 21 datasets.

The cross-strand junction sites of cscR-2-1 are illustrated by 101 RNA-seq reads obtained from 21 datasets combined. Read 2 of these strand-specific PE reads were divided into 2 fragments, which were aligned to the two strands of the genome.

Figure S3

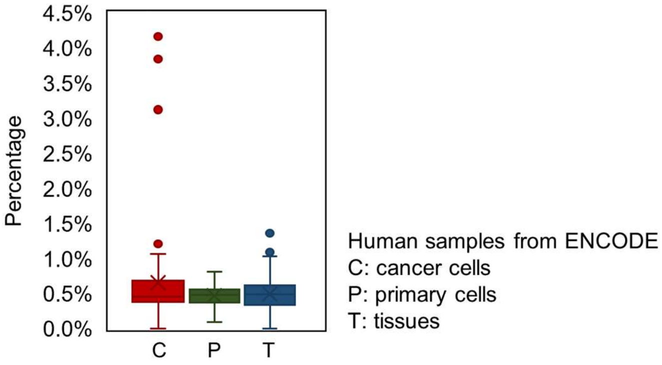

Figure S3. Assessment of the cscRNAs potentially resulted from mapping errors.

For each of the 54 samples of cancer cell lines, 109 samples of primary cells, and 108 samples of normal tissues, the percentages of the cscRNAs of which the sequences around the cross-strand junction sites showed high similarity to the reference genome were counted. The median value is shown as the line and the average as the cross. Source data are provided as a Source Data file.

Figure S4

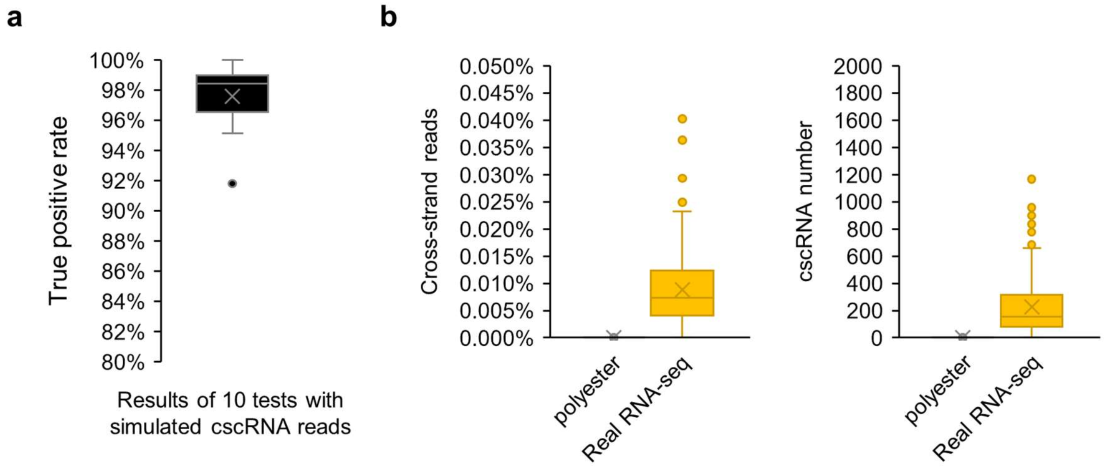

Figure S4. Assessment of the cscMap pipeline with simulated data.

(a) For the sensitivity of cscMap to the cross-strand junction reads, the real RNA-seq data in ENCODE was supplied with simulated cross-strand junction reads from randomly designed cscRNAs. The tests were performed for 10 times, and the recovery rates of these simulated cross-strand junction reads by cscMap were summarized as the box plot. The median value is shown as the line and the average as the cross. Source data are provided as a Source Data file.

(b) We used Polyester to generate the junction reads from the same DNA strand but unmappable to the reference transcriptome or fusion reads between sequences from different chromosomes. These reads were supplemented into the real RNA-seq datasets as inputs of cscMap. This procedure was repeated for 10 times, and the ratios of the cross-strand junction reads or numbers of the cscRNAs identified by cscMap were summarized as box plots, which indicate the general false discovery rates. The median value is shown as the line and the average as the cross.

Figure S5

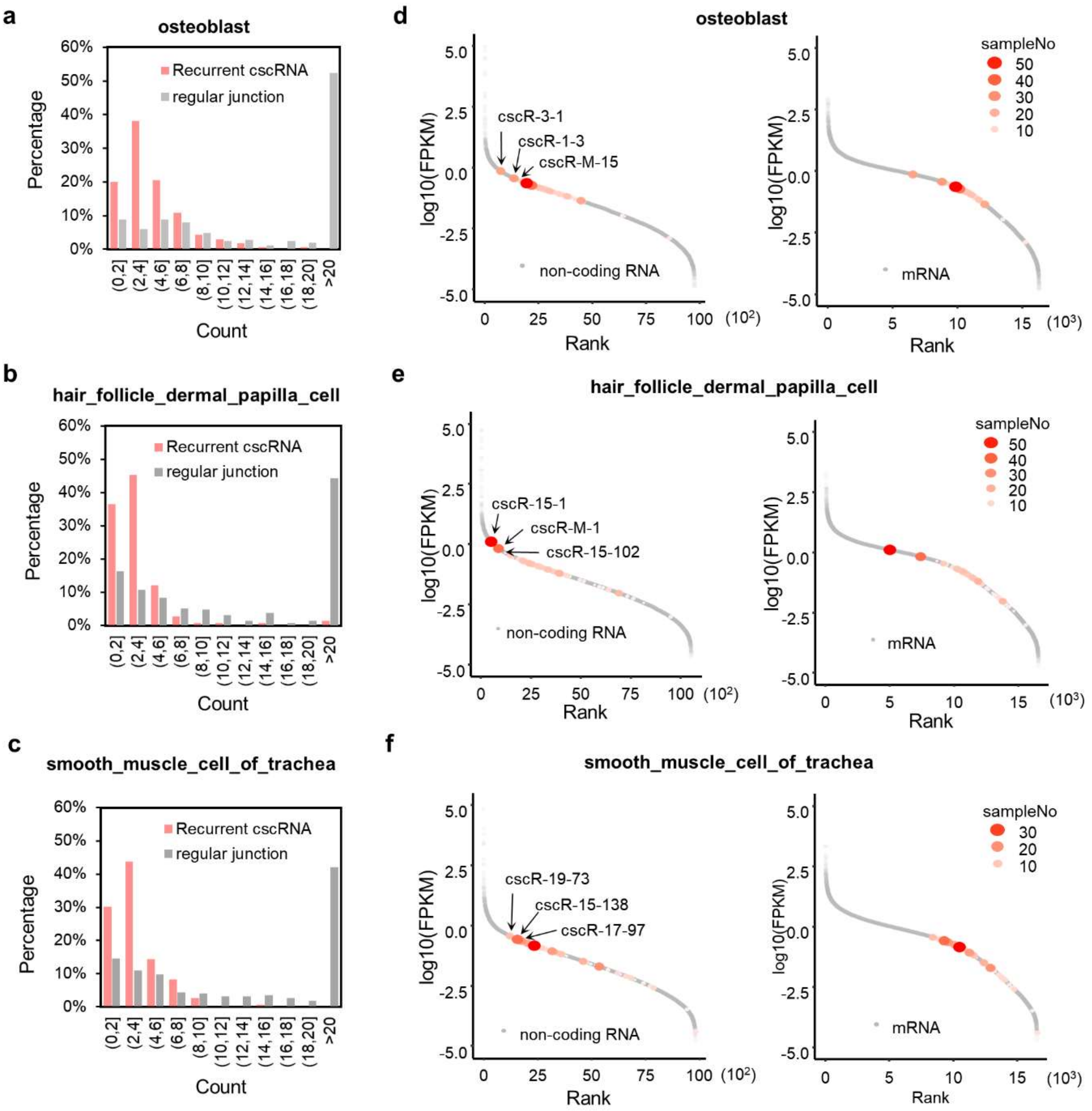

Figure S5. Expression levels of the cscRNAs in 3 samples.

(a-c) Distributions of the RNA-seq read counts, in 3 samples, on the cross-strand junction sites of the recurrent cscRNAs or 10000 randomly selected regular exon-exon junction sites.

(d-f) The FPKMs of the cscRNAs on the background of all the annotated long non-coding RNA (left) or mRNA (right) species, in 3 samples as indicated on the plots. The top 3 cscRNAs in each sample were marked on the plot. The size of each dots indicates the number of samples in which the cscRNA is expressed.

Figure S6

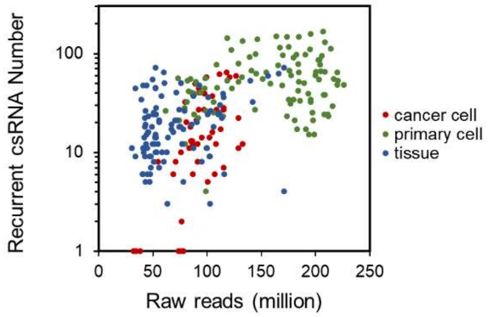

Figure S6. Expression patterns of the recurrent cscRNAs.

The numbers of the recurrent cscRNAs expressed in each of the 271 samples from ENCODE.

Figure S7

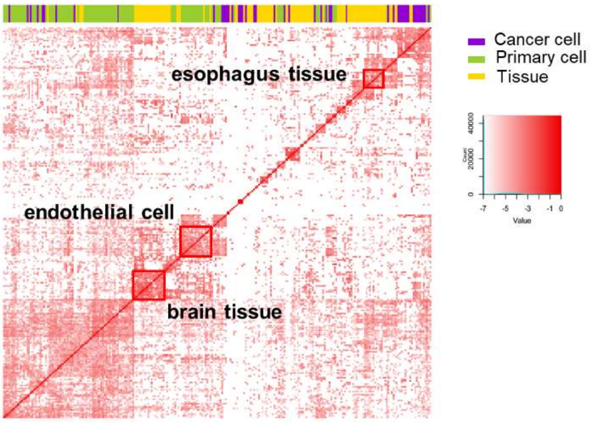

Figure S7. Similarities among the 271 human samples based on the shared recurrent cscRNAs.

Similarity matrix showing the log2 Ochiai values based on the recurrent cscRNAs shared by the 271 human samples from ENCODE. purple: cancer cells; green: primary cells; yellow: normal tissues.

Figure S8

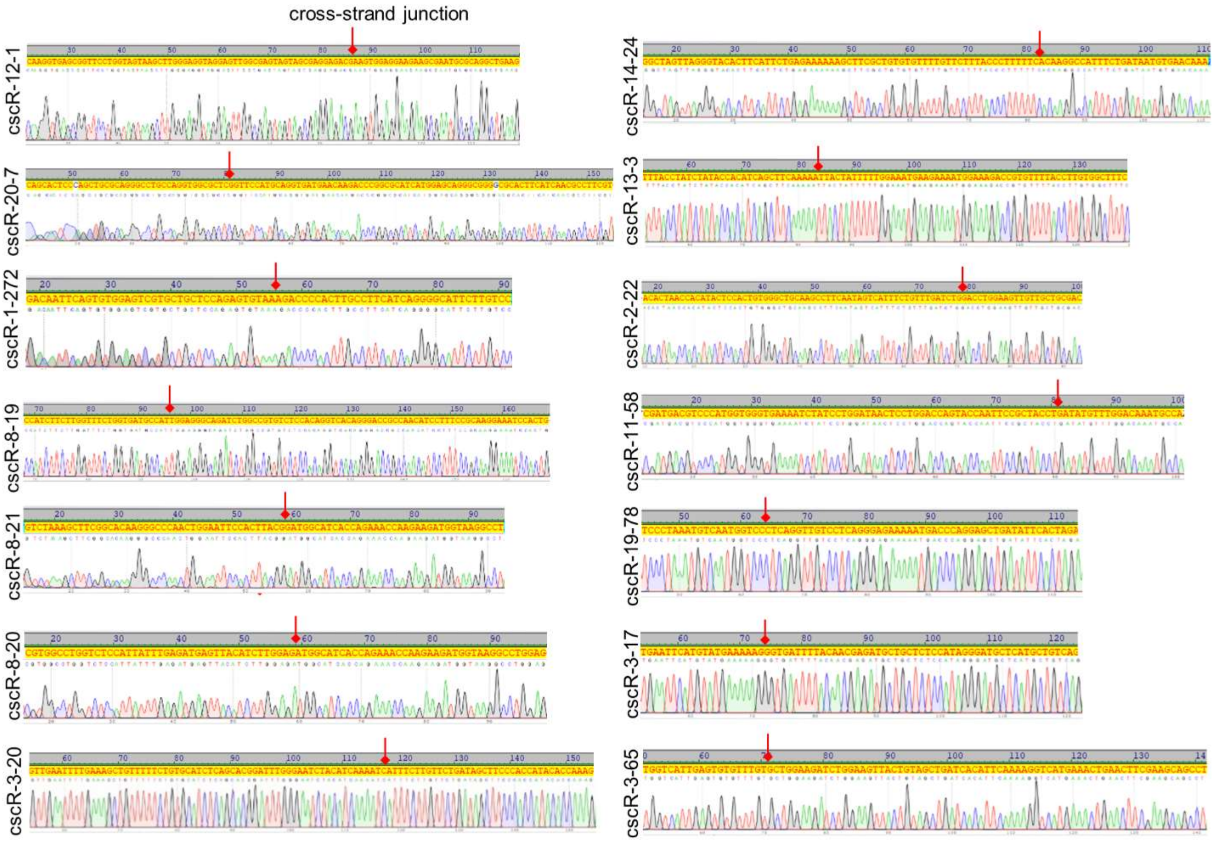

Figure S8. The Sanger sequencing results of 14 cscRNAs.

Supplementary to Fig. 3. The sequences covering the cross-strand junction sites of the cscRNAs were amplified by PCR, followed by Sanger sequencing. The cross-strand junction sites are marked on the sequences.

Figure S9

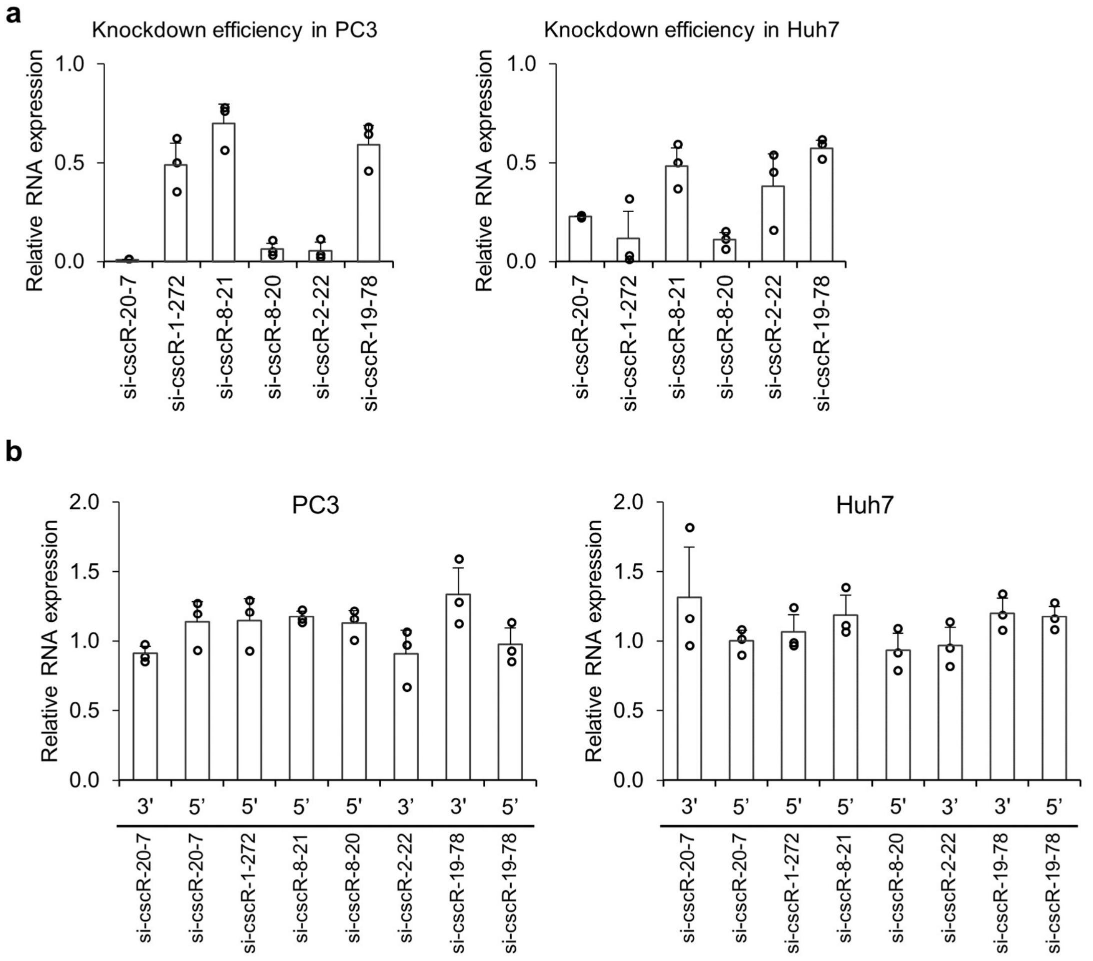

Figure S9. Knockdown efficiencies of the cscRNAs.

(a, b) Relative expression levels of the cscRNAs (a) and the 3' or 5' parental transcripts of the cscRNAs (b) measured by RT-qPCR in PC3 and HUH7 cells, after siRNA-mediated knockdown of the cscRNAs, accordingly. The assays in panel b used PCR primers targeting the regions of the parental transcripts that are outside of the cscRNAs, i.e., upstream of the 3' parental transcripts or downstream of the 5' parental transcripts. Some of these regions were undetectable by pPCR, thereby being absent on the plots (b). Beta-actin was used as a house-keeping gene for normalization. Data show means ±SD of three biological replicates. Source data are provided as a Source Data file.

Figure S10

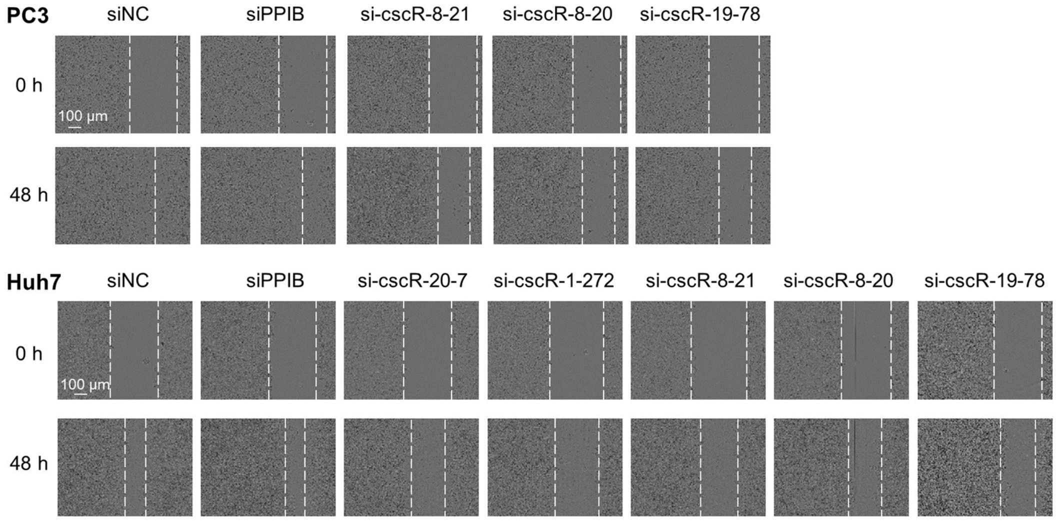

Figure S10. Representative images of the wound healing assays upon cscRNA knockdown. Supplementary to Fig. 4b. Upon siRNA-mediated knockdown, wound healing assay showing scratched area being reoccupied by the PC3 and Huh7 cells migrating from the two sides.

Figure S11

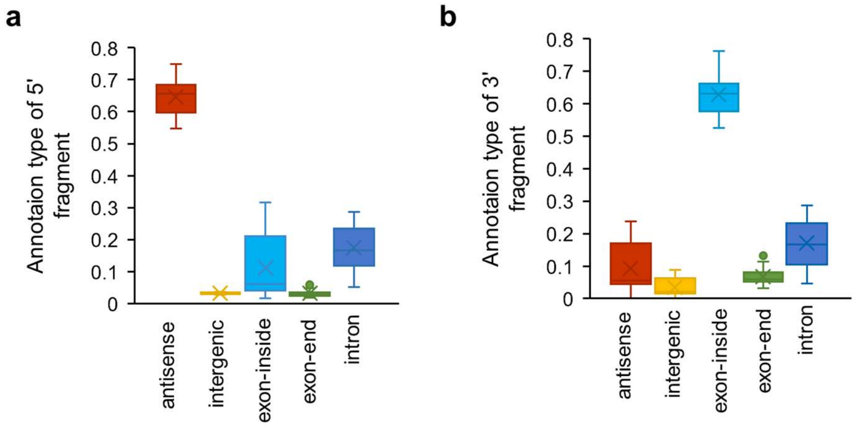

Figure S11. Annotations of the cross-strand junction sites of the cscRNAs in mouse samples.

Box plots showing the proportions of the cscRNAs, in each of the mouse samples, categorized according to the genomic annotations of the 5' or 3' junction sites. antisense: the antisense strand of an annotated gene; intergenic: intergenic region of the genome not being annotated to any gene; exon-inside: inside of an exon region; exon end: the 5' cross-strand junction site being the 3' end of an exon or the 3' junction end being the 5' end of an exon; intron: inside of an annotated intron. The median value is shown as the line and the average as the cross.

Figure S12

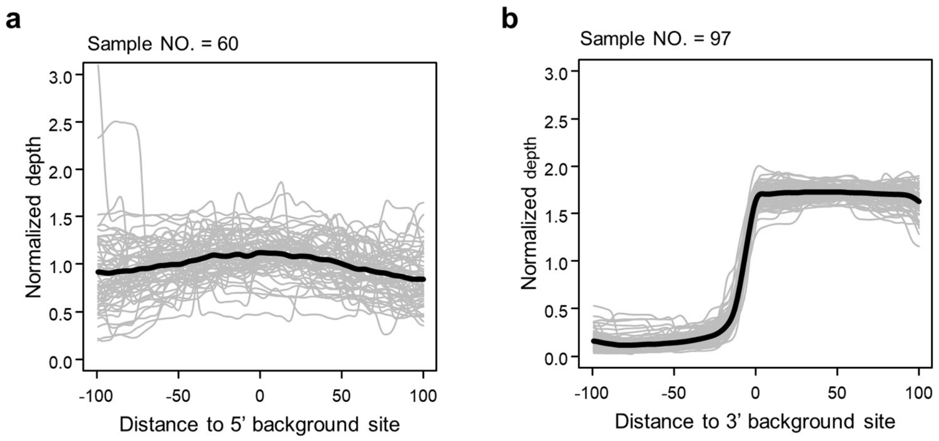

Figure S12. Read densities in the canonical antisense and intron-exon junction regions. Supplementary to Fig. 6c and e. Average read depths along the canonical antisense regions (a) and around the regular intron-exon junctions (b). For each sample (represented by a grey line), the value of each position was calculated by taking the average of the read depths of 200 randomly selected regions in the particular sample. Such analyses were performed for the 45 samples used in Fig. 6c (a) or the 55 samples in Fig. 6e (b). Finally, the average of the 45 or 55 samples on each position was shown as the thick black line.

Figure S13

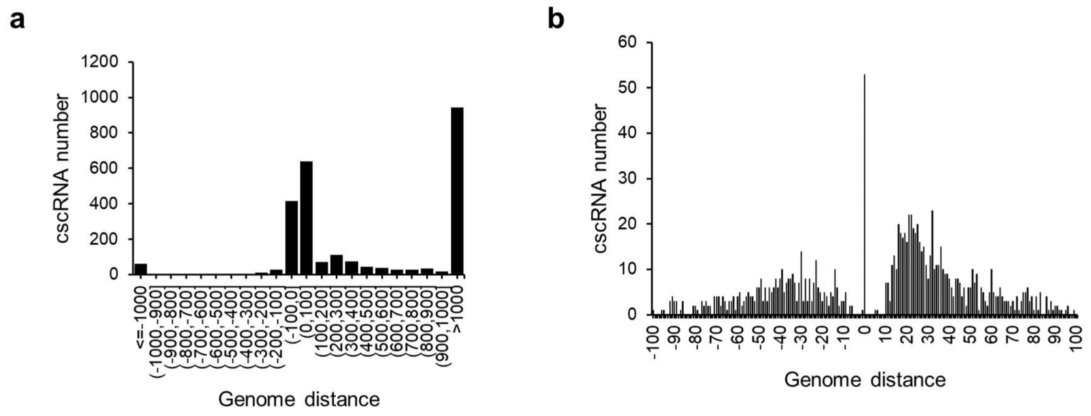

Figure S13. Relative genomic locations of the 5' and 3' cross-strand junction sites of the recurrent cscRNAs.

(a) Distributions of the genomic distances between the 5' and 3' cross-strand junction sites of the recurrent cscRNAs. The positive values indicate 5' junction sites at the up-stream region of the 3' junction sites, and the negative values indicate the opposite.

(b) Zoom-in of panel a, showing the relative distributions of the 5' and 3' cross-strand junction sites that are close to each other (within 100 nt).

Figure S14

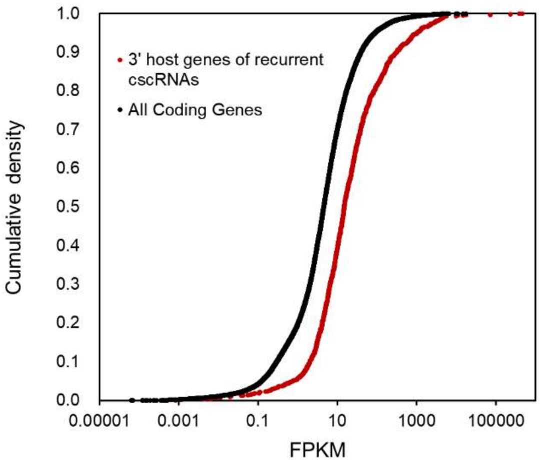

Figure S14. Expressions of the cscRNA host genes.

The maximum FPKM values of all the protein coding genes in the ENCODE datasets are shown in black dots and the host genes of the cscRNAs shown in red dots.

Supplementary Table 1. Primer sequences for the PCR assays.

ID	Forward primer (5’-3’)	Reverse primer (5’-3’)
cscR-12-1	CAAGGTGAGCGGTTCCTGGTA	GCTTCAGCCTGCGCATTC
cscR-20-7	CCCTGGAGAAGATCCTAGC	CGAGGAGCAGTTCTCATT
cscR-1-272	GACAATTCAGTGTGGAGTCGTG	GGACAAGAATGCCCCTGATG
cscR-8-19	CCATCTTCTTGGTTTCTGGTGA	CAGTGGATTTCCTTGCGGAA
cscR-8-21	GTCTAAAGCTTCGGCACAAGGG	AGGCCTTACCATCTTCTTGGTT
cscR-8-20	TCGTGGCCTGGTCTCCATTATTT	CTCCAGGCCTTACCATCTTCTTG
cscR-2-1	CGGTCACTGCACTCTCAG	TCTGCTCCAGCGAACAAC
cscR-19-8	CTCGTCCTTCCGGGTATCAG	TTGTTCATCATCAGCACAGGC
cscR-3-20	CTTTGGTGTATGGTGGGAAGC	GTTGAATTTTGAAAGCTGTTTTTC TGT
cscR-14-24	TTGTTCACATTATCAGAAATGGCCT	CGGCTAGTTAGGGTACACTTCAT
cscR-13-3	TACCTATCTATACCACATCAGCTTC	GAAAGCCACAAGGTAAAACACG
cscR-2-22	GTCGCAGCAACAACTTCCAG	ACACTAACCACATACTCCACTGT
cscR-11-58	GGCATTTGTCCAAACATATCAGGT	CGATGACGTCCCATGGTGG
cscR-19-78	CTAGTGAATATCAGCTCCTGGGTC	TCCCTAAATGTCAATGGTCCCTC
cscR-3-17	TGAATTCATGTATGAAAAAGGGTGA	CTGACAGCATGAGCATCCCT
cscR-3-65	TGGTCATTGAGTGTGTTTGTGCT	AGGCTGCTTCGAAGTTCAGTT
LMNA	ATGAGGACCAGGTGGAGCAGTA	ACCAGGTTGCTGTTCCTCTCAG
PPIB	AACGCAGGCAAAGACACCAACG	TCTGTCTTGGTGCTCTCCACCT
ACTB	CACCATTGGCAATGAGCGGTTC	AGGTCTTTGCGGATGTCCACG

Supplementary Table 2. siRNA sequences used in the study.

Oligos	Sense (5’-3’)	Antisense (5’-3’)
cscR-12-1	GAGACGAAGUGGAGGAAGAAG	UCUUCCUCCACUUCGUCUCC
cscR-20-7	AGGUGGCGCUCGGUUCCAUGC	AUGGAACCGAGCGCCACCUG
cscR-1-272	CCAGAGUGUAAAGACCCCACU	UGGGGUCUUUACACUCUGGA
cscR-8-19	GAUCUGCCCUCCAUGGCAUCA	AUGCCAUGGAGGGCAGAUCU
cscR-8-21	CCACUUACGGAUGGCAUCACC	UGAUGCCAUCCGUAAGUGGA
cscR-8-20	CUUGGAGAUGGCAUCACCAGA	UGGUGAUGCCAUCUCCAAGA

cscR-2-1	CACAAACUGCAGCGCCUGAUG	UCAGGCGCUGCAGUUUGUGG
cscR-19-8	UGAAGCGUGUCAGGCGUGUGC	ACACGCCUGACACGCUUCAC
cscR-3-20	GAACAAGAAAUGAUUUUGAUG	UCAAAAUCAUUUCUUGUUCU
cscR-14-24	GAAAUGGCCUUGUGAAAAAGG	UUUUUCACAAGGCCAUUUCU
cscR-13-3	CUUCAAAAAUUACUAUUUUUG	AAAAUAGUAAUUUUUGAAGCU
cscR-2-22	UCCAGGUCCAGAUCAAACAGA	UGUUUGAUCUGGACCUGGAA
cscR-11-58	CAUAUCAGGUAGCGGAAUUGG	AAUUCCGCUACCUGAUAUGU
cscR-19-78	CAACCUGAGGGACCAUUGACA	UCAAUGGUCCCUCAGGUUGU
cscR-3-17	UUGUAAAAUCACCCUUUUUCA	AAAAAGGGUGAUUUUACAACG
cscR-3-65	GUGUGUUUGUGCUGGAAGAUC	UCUUCCAGCACAAACACACUC
Host 8-21	UGAAGAUCACUGUAAAUCCAU	GGAUUUACAGUGAUCUUCAG
Host 8-20	UGAAAUCCCUCAGGGCUUCAC	GAAGCCCUGAGGGAUUUCAU
Host 2-22	UAGACUUGCUCCCAGGCACAG	GUGCCUGGGAGCAAGUCUAC
PPIB	CCUACGAAUUGGAGAUGAAGA	UCUUCAUCUCCAAUUCGUAGG
NC	UUCUCCGAACGUGUCACGU	ACGUGACACGUUCGGAGAA
