# Supplementary material for: Identification of the cross-strand chimeric RNAs generated by fusions of bi-directional transcripts
Source: Nat Commun. 2021 Jul 30;12:4645. doi: 10.1038/s41467-021-24910-2 (PMC8324879; doi:10.1038/s41467-021-24910-2)
Supplement: Supplementary file 5 — Description of additional supplementary files [file 41467_2021_24910_MOESM5_ESM.docx]

Description of additional supplementary information

Title: Supplementary Table 1.

Description: Primer sequences for the PCR assays.

Title: Supplementary Table 2.

Description: siRNA sequences used in the study.

Title: Supplementary Data 1.

Description: Information of the datasets used in the present study.

Title: Supplementary Data 2.

Description: Information of the cscRNAs identified in human samples.

Title: Supplementary Data 3.

Description: Information of the cscRNAs identified in mouse samples.

Title: Supplementary Software.

Description: The scripts of cscMap and a demo dataset.
