# Supplementary material for: Identification of the cross-strand chimeric RNAs generated by fusions of bi-directional transcripts
Source: Nat Commun. 2021 Jul 30;12:4645. doi: 10.1038/s41467-021-24910-2 (PMC8324879; doi:10.1038/s41467-021-24910-2)
Supplement: Supplementary file 7 — Reporting Summary [file 41467_2021_24910_MOESM7_ESM.pdf]

Corresponding author(s): Xuerui Yang

Last updated by author(s): Jul 3, 2021

Reporting Summary

Nature Research wishes to improve the reproducibility of the work that we publish. This form provides structure for consistency and transparency in reporting. For further information on Nature Research policies, see our Editorial Policies and the Editorial Policy Checklist.

Statistics

For all statistical analyses, confirm that the following items are present in the figure legend, table legend, main text, or Methods section.

n/a	Confirmed	
☐	☒	The exact sample size (*n*) for each experimental group/condition, given as a discrete number and unit of measurement
☐	☒	A statement on whether measurements were taken from distinct samples or whether the same sample was measured repeatedly
☐	☒	The statistical test(s) used AND whether they are one- or two-sided<br>*Only common tests should be described solely by name; describe more complex techniques in the Methods section.*
☒	☐	A description of all covariates tested
☐	☒	A description of any assumptions or corrections, such as tests of normality and adjustment for multiple comparisons
☐	☒	A full description of the statistical parameters including central tendency (e.g. means) or other basic estimates (e.g. regression coefficient) AND variation (e.g. standard deviation) or associated estimates of uncertainty (e.g. confidence intervals)
☐	☒	For null hypothesis testing, the test statistic (e.g. *F*, *t*, *r*) with confidence intervals, effect sizes, degrees of freedom and *P* value noted<br>*Give P values as exact values whenever suitable.*
☒	☐	For Bayesian analysis, information on the choice of priors and Markov chain Monte Carlo settings
☐	☒	For hierarchical and complex designs, identification of the appropriate level for tests and full reporting of outcomes
☒	☐	Estimates of effect sizes (e.g. Cohen's *d*, Pearson's *r*), indicating how they were calculated

Our web collection on statistics for biologists contains articles on many of the points above.

Software and code

Policy information about availability of computer code

Data collection	No software was used for collection of the public data.
Data analysis	cscMap is a bioinformatics pipeline to search for the RNA chimeras resulted from fusions of the transcripts encoded by the two opposite DNA strands. The cscMap pipeline has been provided as a supplementary file and deposited in github (https://github.com/xryanglab/cscMap). Other softwares used in the present study include tophat 2.0, bowtie 2.0, samtools, bedtools and python 2.7.

For manuscripts utilizing custom algorithms or software that are central to the research but not yet described in published literature, software must be made available to editors and reviewers. We strongly encourage code deposition in a community repository (e.g. GitHub). See the Nature Research guidelines for submitting code & software for further information.

Data

Policy information about availability of data

All manuscripts must include a data availability statement. This statement should provide the following information, where applicable:

- Accession codes, unique identifiers, or web links for publicly available datasets
- A list of figures that have associated raw data
- A description of any restrictions on data availability

The authors declare that the main data supporting the findings of this study are available within the article and its Supplementary Information files. Most of the human rRNA-depleted total RNA-seq datasets were obtained from the ENCODE project. The datasets from other species were obtained from the GEO database. The details of all the datasets used in the present study are provided in Supplementary Data 1.

Field-specific reporting

Please select the one below that is the best fit for your research. If you are not sure, read the appropriate sections before making your selection.

☒ Life sciences ☐ Behavioural & social sciences ☐ Ecological, evolutionary & environmental sciences

For a reference copy of the document with all sections, see nature.com/documents/nr-reporting-summary-flat.pdf

Life sciences study design

All studies must disclose on these points even when the disclosure is negative.

Sample size	Total RNA-seq datasets include 271 human samples and 20 mouse samples from the ENCODE project, which offers advantages of high sequencing depths, large sample size, tissue type variety, and strand-specific sequencing with long paired-end reads. The datasets from other species were obtained from the GEO database, including zebrafish (GSE73615 and GSE74929), yeast (GSE110413 and GSE99170), C. elegans (GSE79375 and GSE79375), fruitfly (GSE83877 and GSE101603), and E. coli (GSE41190). Datasets from some human samples were also downloaded from the GEO database, including MCF7 cells (GSE94372 and GSE89888), PC3 cells (GSE65112 and GSE48230), and lung tissues (GSE60052 and GSE52248). The whole genome DNA-seq data was obtained from the GEO database (GSE48216). The dataset includes 9 breast epithelial cell lines (MCF10A, MCF10F, MCF12A, HCC1143, HCC1143BL, HCC1187, T4, T47D, T47D_KBluc). The analyses were done on a per-sample basis. The sample sizes are sufficient for all the analyses.
Data exclusions	No data was excluded from the analyses.
Replication	All the experimental data were obtained with at least 3 biological replicates. All attempts at replication were successful.
Randomization	For all the experiments, the samples were randomly allocated into groups.
Blinding	Blinding was not relevant as no direct comparison between experimental groups was done. The study is mainly analysis of the public data.

Reporting for specific materials, systems and methods

We require information from authors about some types of materials, experimental systems and methods used in many studies. Here, indicate whether each material, system or method listed is relevant to your study. If you are not sure if a list item applies to your research, read the appropriate section before selecting a response.

Materials & experimental systems

n/a	Involved in the study
☐	☒ Antibodies
☐	☒ Eukaryotic cell lines
☒	☐ Palaeontology and archaeology
☒	☐ Animals and other organisms
☒	☐ Human research participants
☒	☐ Clinical data
☒	☐ Dual use research of concern

Methods

n/a	Involved in the study
☒	☐ ChIP-seq
☒	☐ Flow cytometry
☒	☐ MRI-based neuroimaging

Antibodies

Antibodies used	Sheep anti-DIG antibody (Roche, 11333089001), Donkey Anti-Sheep IgG H&L fluorophore-conjugated secondary antibody (Abcam, ab150180)
Validation	Sheep anti-DIG antibody was validated by the manufacturer and used in at least 25 peer-reviewed research articles.

Eukaryotic cell lines

Policy information about cell lines

Cell line source(s)	All the cell lines (K562, MCF7, NCI-H460, A549, HUH7, PC3) were from ATCC.
Authentication	All the cells were authenticated with the methods provided by ATCC, including Karyotyping, DNA barcoding, and PCR assays with species-specific primers.
Mycoplasma contamination	All the cells were tested for mycoplasma contamination every 3 months, and the results have been negative.

Commonly misidentified lines<br>(See ICLAC register)	No commonly misidentified cell lines were used in the study.
